# Identification of Volatile and Semi-Volatile Compounds in Polymeric Coatings Used in Metal Cans by GC-MS and SPME

**DOI:** 10.3390/ma14133704

**Published:** 2021-07-02

**Authors:** Patricia Vázquez-Loureiro, Antía Lestido-Cardama, Raquel Sendón, Julia López-Hernández, Perfecto Paseiro-Losada, Ana Rodríguez-Bernaldo de Quirós

**Affiliations:** Department of Analytical Chemistry, Nutrition and Food Science, Faculty of Pharmacy, University of Santiago de Compostela, 15782 Santiago de Compostela, Spain; patriciavazquez.loureiro@usc.es (P.V.-L.); antia.lestido@usc.es (A.L.-C.); raquel.sendon@usc.es (R.S.); julia.lopez.hernandez@usc.es (J.L.-H.); perfecto.paseiro@usc.es (P.P.-L.)

**Keywords:** potential migrants, polymeric coatings, GC-MS, SPME

## Abstract

Polymeric coatings are used as a protective layer to preserve food or beverage quality and protect it from corrosion and avoid a metallic taste. These types of materials can contain some chemicals that are susceptible to migrate to food and constitute a risk for consumers’ health. This study is focused on the identification of volatile and semi-volatile low molecular weight compounds present in polymeric coatings used for metal food and beverage cans. A method based on solid–liquid extraction followed by gas chromatography–mass spectrometry (GC-MS) was optimized for the semi-volatile compounds. Different solvents were tried with the aim of extracting compounds with different polarities. Furthermore, a method based on solid-phase microextraction (SPME) in headspace (HS) mode and gas chromatography coupled with mass spectrometry (HSSPME-GC-MS) was developed for the identification of potential volatile migrants in polymeric coatings. Some parameters such as extraction time, equilibrium temperature, or the type of fiber were optimized. Different compounds, including aldehydes such as octanal or nonanal, alcohols such as α-terpineol or 2-butoxyethanol, ethers, alkenes, or phthalic compounds, among others, were identified and confirmed with analytical standards both via SPME analysis as well after solvent extraction.

## 1. Introduction

Migration of components from food contact material to food is a matter of concern from the food safety point of view. Special attention has been paid to low molecular weight compounds and particularly to unknown compounds. Their identification is a current challenge in the food packaging field [1].

Different materials have traditionally been used in food packaging including glass, metals, paper, paperboards, and plastics. Marsh et al. [2] reported some advantages and disadvantages of these typical materials used in beverage packaging, such as the susceptibility to breakages or the heavy weight in the case of glass. Metal cans are widely used, and they have several advantages over other materials as they are able to tolerate high temperature and pressure conditions [3]. Polymeric coatings are used as functional barriers between food and metal cans. They preserve the quality of food in terms of flavor, odor, and color, as well as extend shelf-life and help the metal can in protecting food from external agents such as light, oxygen, and microorganisms, and facilitate transport and storage of the canned food.

Beverage packaging often combines several materials to exploit these properties. Multilayer systems, new approaches based on active or intelligent packaging or materials with lower environmental impacts are in development [2].

Beverage cans are one of the most used multilayer packaging materials, made of aluminum with an inner epoxy resin coating to prevent direct contact between food or beverage and the aluminum surface [4]. Final coatings are obtained by the addition of components such as cross-linkers, solvents, pigments, anti-foaming agents, adhesion promoters, resins, and surfactants [5]. During the polymerization process, side reactions can occur, and linear or cyclic byproducts may be formed. These unknown chemicals may migrate into food resulting in consumer exposure [6].

Epoxy resins are commercially used in coatings because of their exceptional adhesion due to the presence of polar hydroxyl and ether groups in their structure [7]. Besides this technical advantage, some drawbacks regarding their safety can be found in the literature; some authors have shown the potential migration of bisphenol A (BPA) from these materials to food [8]. Epoxy monomers such as bisphenol A-diglycidyl ether (BADGE) have been extracted from epoxy resins [9] and found in food simulants after migration assays [10], and other BADGE-based derivatives from epoxy coatings were also identified by Schaefer et al. [11].

Commission Regulation (EU) No. 10/2011 [12] established specific rules for plastic materials intended to come in contact with food. Currently, there is no specific European legislation for coatings. Both intentionally added substances (IAS) and non-intentionally added substances (NIAS) such as impurities, reaction byproducts, and degradation products can migrate into food, and they should be evaluated.

These migrants may also be oligomers, prepolymers, catalyst, reaction accelerators, epoxidized edible oils, esters, waxes, lubricants, metals, etc. [13,14]. The migration of these chemicals from packaging to food and beverage is one of the main concerns of food safety authorities.

Non-targeted methods using LC-MS or GC-MS are being widely employed for the identification of potential migrants in food packaging. Both techniques provide essential and complementary information necessary for a complete characterization of packaging materials. In GC-MS analysis, the use of commercial libraries helps the identification, although in the case of NIAS, they usually are not present in the databases. Bradley et al. [15] carried out an analysis via headspace GC-MS and a solvent extraction with acetonitrile followed by GC-MS to determine volatile compounds in epoxy phenolic can coatings. The authors detected bisphenol A, used as a starting substance in the manufacturing of the coating. More recently, Omer et al. [16] used GC-MS with different ionization sources, namely electron ionization (EI) and atmospheric pressure chemical ionization (APCI), and different mass spectrometers, specifically quadrupole, time-of-flight, and orbitrap, to investigate potential migrants in polyester–polyurethane lacquers. Several cyclic oligoester tetramers were identified in the two lacquers tested. In another study reported in the literature, GC-MS and highly accurate mass spectrometry was used for the analysis of bisphenol A alternative food-contact metal can coatings. Cyclic polyester oligomers from polyester-based coatings and bisphenol-type compounds, including tetramethyl bisphenol F, tetramethyl bisphenol F diglycidyl ether, and bisphenol F, among others, were identified [3].

The aim of this work was to develop a screening method for the identification of volatile compounds in polymeric coatings of metal cans for beverage packaging. For that purpose, a method based on solid–liquid extraction followed by gas chromatography–mass spectrometry (GC-MS) and a method based on solid-phase microextraction in headspace mode and gas chromatography coupled with mass spectrometry (HSSPME-GC-MS) were optimized.

## 2. Materials and Methods

### 2.1. Sample Description and FTIR Characterization

A total of ten beverages packed in metal cans were bought in local supermarkets in Santiago de Compostela (Spain). All of them were two-piece cans. The internal surface of metal cans is often coated with a polymeric coating (with a thickness of about 2 μm) to preserve food and avoid metal corrosion. The thickness of the samples analyzed (metal + coating) is provided in Table 1. The thickness of the packaging was measured with a manual digital micrometer (Mitutoyo-Japan, Kanagawa, Japan). The polymeric coatings were analyzed by using an attenuated total reflectance FTIR spectrometer and were identified using the KnowItAll^®^ 17.4.135.B IR Spectral Libraries of Polymers and Related Compounds (Bio-Rad Laboratories, Inc., Hercules, CA, USA). 

The pH of the beverage samples ranged between 2.56 and 6.60. A brief overview of the samples is presented in Table 1; and a more detailed description of the samples used in this study was described by Lestido-Cardama et al. [17].

### 2.2. Sample Treatment

#### 2.2.1. Solvent Extraction Procedure

Samples were opened, emptied, and washed with warm water before analysis. Cans were cut into small pieces (approximately 0.5 cm^2^), then 0.8 g were weighted in a vial and 5 mL of methanol was added and afterward the vial was hermetically sealed. The extraction was performed in an oven at 70 °C for 24 h. One aliquot was then removed with a 0.22 µm polytetrafluoroethylene (PTFE)-membrane filter and analyzed via GC-MS.

#### 2.2.2. SPME Procedure

An SPME holder for manual sampling and commercial fibers was purchased from Supelco (Bellefonte, PA, USA). Fibers with different coating materials were tested: a divinylbenzene-Carboxen-polydimethylsiloxane (DVB/PDMS/CAR) fiber with 50–30 µm thickness and a Carboxen-polydimethylsiloxane (CAR-PDMS) fiber with 100 µm thickness. Prior to use they were conditioned by inserting them into the GC injector according to the supplier’s instructions: for 1 h at 270 °C and 0.5 h at 250 °C, respectively.

For each experiment, 0.8 g of each sample, previously cut into small pieces (approximately 0.5 cm^2^) were weighted into a 20 mL headspace vial and sealed with a PTFE-faced silicone septum (Cromlab, Barcelona, Spain). The SPME fiber was put into the vial, and this was heated at 100 °C for 30 min. The fiber was then desorbed into the GC injector for 10 min at 200 °C. The compounds absorbed by the fiber were separated via gas chromatographic analysis and identified using the MS detector operating in the described conditions below.

### 2.3. Reagents and Analytical Standards

Acetonitrile HPLC grade, methanol, and hexane GC-MS grade were supplied by Merck (Darmstadt, Germany). Ethanol for analysis was supplied by Merck (Darmstadt, Germany). The 2-butoxyethanol, spectrophotometric grade with a purity of ≥99.0%, 2,2-dimethyl-1,3-propanediol with a purity of 99.0%, octanal with a purity of 99%, D-Limonene, ε-Caprolactam; 2,6-Di-tert-butyl-1,4-benzoquinone with a purity of 98.0%, diethyl phthalate with a purity of 99.5%, benzophenone with a purity of ≥99.0%, benzoic acid with a purity of 99.5%, vanillin with a purity of 99.0%, 2,4-ditertbutylphenol with a purity of 99.0%, and α-terpineol were purchased from Sigma Aldrich (Steinheim, Germany). Nonanal with purity of 98.7% was purchased from Supelco (Bellefonte, PA, USA). The 2-phenoxyethanol with purity of ≥99.0% was supplied by Fluka (Seelze, Germany). Working solutions were prepared by diluting different amounts of the stock standard solution in methanol.

### 2.4. GC-MS Conditions for Solvent Extraction Samples

A Trace 1300 gas chromatograph equipped with a programmed split/splitless injector, a 1310 autosampler, and an ISQ LT Single Quadrupole mass spectrometer (Thermo Electron Corp., Madison, WI, USA) were used to perform the GC analyses. The separation was performed on a Rxi-5Sil MS (30 m × 0.25 mm × 0.25 μm) column from Restek (Bellefonte, PA, USA). The operating conditions were the following: the injector temperature was 300 °C and the temperature of the transfer line of the detector was 300 °C. The oven temperature was set as follows: initially the temperature was set at 40 °C for 2 min, then increased at a rate of 9 °C/min until 300 °C and held for 12 min. Injection was performed in splitless mode, and the injection volume was 1 µL. The carrier gas was helium with a flow rate of 1 mL/min. The mass spectrometer was operated in electron impact ionization mode with a full scan range between 20 and 500 *m*/*z*. 

Data analysis was performed using Xcalibur version 4.1 and the NIST/EPA/NIH 11 mass spectral library (version 2.0) and Wiley Registry TM 8th edition database were used for identification.

### 2.5. GC-MS Conditions for SPME Analysis

A Thermo Finnigan Trace GC gas chromatograph and a Finnigan Trace DSQ mass selective detector (Thermo Scientific, Waltham, MA, USA) were used to perform all GC analyses. For SPME analyses, an Rxi-624Sil MS (30 m × 0.25 mm × 1.40 µm) column from Restek (Bellefonte, PA, USA) was used, and the separation of compounds was performed under the following operating conditions: the injector temperature was set at 200 °C and the transfer line temperature was 250 °C. The ramp temperature was set from 45 to 250 °C. The mass spectrometer operated in full scan mode (between 20 and 500 *m*/*z*).

Data analysis was performed using Xcalibur version 2.0.7 and the NIST/EPA/NIH 11 mass spectral library (version 2.0) and Wiley Registry TM 8th edition database were used for detection and identification.

In order to estimate the toxicity of the identified compounds, an in silico method, namely Cramer rules were applied. For that, the software Toxtree was used [18]. According to Cramer rules, substances are classified based on their chemical structure into Class I (low toxicity), Class II (intermediate toxicity) and Class III (high toxicity). Thus, Class I comprises substances with simple chemical structures such as common carbohydrates, acyclic aliphatic hydrocarbons, and so on. Class II includes substances that possess structures that are less innocuous than those of Class I but do not contain substances with structural features that suggest toxicity like substances of Class III. Examples of Class II substances are common components of food, substances containing no functional groups other than alcohol, aldehyde, acid, ester, etc. Class III includes substances with chemical structures that may suggest significant toxicity or contain reactive functional groups. Examples of substances belonging to this Class are certain benzene derivatives, certain heterocyclic substances, etc. [19].

## 3. Results and Discussion

### 3.1. Solvent Selection for Can Extraction

**Figure 1:**
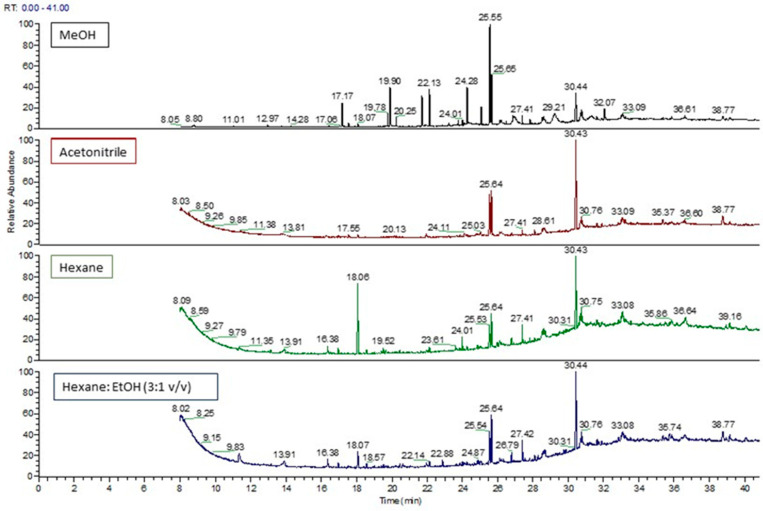
Chromatograms of sample BC04 extracted with different solvents.

Different solvents were tested with the aim to extract compounds with different polarities present in the coating of metal cans. Samples were extracted under different conditions, both methanol and acetonitrile for 24 h at 70 °C, hexane for 4 h at 60 °C, and a mixture of hexane and ethanol (3:1 *v*/*v*) for 24 h at 20 °C. In Figure 1, chromatograms obtained after extraction with different solvents are shown. Methanol was the solvent selected for extraction because more peaks were detected and identified. Table 2 lists the compounds detected after extraction with different solvents. As the analyzed samples were already in contact with the drink, some of the identified compounds may have their origin in food. On the other hand, it is interesting to note that flavorings are commonly used in these beverages, thus some of the detected compounds in the samples are authorized as food flavorings in the European Union [20]. These compounds are indicated in the table with their corresponding Flavis Number (FL No.). Some of them are, for example, benzoic acid methyl ester and caprylic acid methyl ester.

Ester compounds were mainly identified in extraction with methanol as a solvent. Some studies show the migration of these types of compounds in cured varnishes used in food packaging [21]. In this work, samples were extracted with ethanol 95% (*v*/*v*). Adipic acid has been reported as a chemical intermediate used in the manufacturing of polyurethane resins [22].

### 3.2. Optimization of SPME Method

In the present work, a method based on solid-phase microextraction in headspace mode and gas chromatography coupled with mass spectrometry (HSSPME-GC-MS) was developed for the identification of potential migrants in polymeric coatings. 

SPME is an easy, cheap, and clean method to use, although there is a for need further optimization in terms of equilibrium of experimental conditions such as heating temperature, extraction time, sample volume, concentration of volatiles, and sample matrix [23]. For that purpose, some parameters such as extraction time, equilibrium temperature, or the type of fiber were optimized.

The effect of extraction temperature, extraction time, and desorption time was evaluated using the fiber DVB-CAR-PDMS.

Firstly, the extraction time was optimized. Different times were tested (10, 30, and 60 min), keeping extraction temperature (40, 70, and 100 °C), equilibration time (2 min), and desorption time (10 min) fixed. Under these conditions the best results were found at 30 and 60 min of extraction because more peaks were identified and with a higher intensity, and there was hardly any difference between the two tested times, therefore, 30 min of extraction was selected. Once the time of extraction was optimized, the temperature was studied ranging from 40 to 100 °C. The difference in the sensitivity and the number of peaks detected was related with the increase of the temperature.

The effects of temperature and extraction time were evident from the chromatograms obtained under the following conditions: 40, 70, and 100 °C for 10, 30, and 60 min. An increase in the peak chromatographic area was found, especially with the less volatile compounds at higher temperatures.

Machiels et al. [24] reported that highly volatile compounds were not affected by desorption time and less volatile compounds needed more time to desorb.

The next parameter that was optimized was the amount of the sample used, which was considered between 0.8 g and 2 g. Finally, the amount 0.8 g was chosen because larger amounts of sample did not lead to higher intensity of the chromatographic peaks.

It is important to get a well-balanced compromise between sensitivity and extraction rate, particularly with respect to the extraction temperature, to achieve a careful optimization of each parameter.

Best results and with the higher peak intensities were obtained for 30 min at 100 °C with 2 min of equilibration time and 10 min of desorption time.

#### Selection of the Type of Fiber

The selection of the fiber and SPME extraction conditions can affect the sensitivity and accuracy of SPME analysis. Park et al. [25] affirmed that using two-phase fibers (Carboxen-PDMS) seems to be more suitable for measuring low molecular weight compounds, whereas three-phase fibers (DVB-Carboxen on PDMS) appeared to be more appropriate for measuring high molecular weight compounds. DVB-CAR-PDMS fiber has shown the best sorption capacity for some compounds such as food packaging contaminants in alcoholic beverages.

In our study, a DVB/PDMS/CAR fiber with 50–30 µm thickness and a CAR-PDMS with 100 µm thickness were tested. Peak areas from decanal, 2-oxepanone and diethylphthalate, which were the most abundant peaks, were compared between both types of fibers, with the first one achieving the higher response. This fact confirms that DVB/PDMS/CAR fiber is more appropriate to separate volatile compounds with higher molecular weight.

### 3.3. Can Coatings Analysis via GC after a Solvent Extraction

GC-MS was used to tentatively identify semi-volatile compounds that could potentially migrate from polymeric coatings. A GC-MS method that covered a wide mass range (from 35 to 500 *m*/*z*) with a suitable gradient of temperatures was used. Samples were injected in splitless mode. Results obtained are shown in Table 3. Only compounds with appropriate direct matching factors (SI) and reverse search matching (RSI) are identified in Table 3. In general, values of 900 or greater are considered an excellent match, 800–900 a good match, and 700–800 a fair match. For those compounds whose identification was not achievable, the most abundant *m*/*z* is specified.

Volatile compounds coming from the beverage were detected. Thus, different esters (e.g., benzoic acid methyl ester, lauric acid methyl ester, etc.) were identified. Esters were reported by Dragone et al. [26] in alcoholic distilled beverages, which contribute to the greatest proportion of the total aroma. The analysis was performed using dichloromethane as an extraction solvent and the compounds were separated on a CP-Wax 52 CB (50 m × 0.25 mm i.d., 0.2 μm film thickness, Chrompack). Ledene, a sesquiterpene hydrocarbon, has also been found in natural products [27]. α-Methyl-δ-oxo-2-phenyl-1,3-dioxolane-2-heptanenitrile has been reported as a precursor of thymol and a carvacrol and eugenol intermediary [28]. Moreover, flavorings authorized in the EU [20], such as α-terpineol and dodecalactone, were identified in different samples. These substances belong to Class III and Class II, according to Cramer rules, respectively.

**Figure 2:**
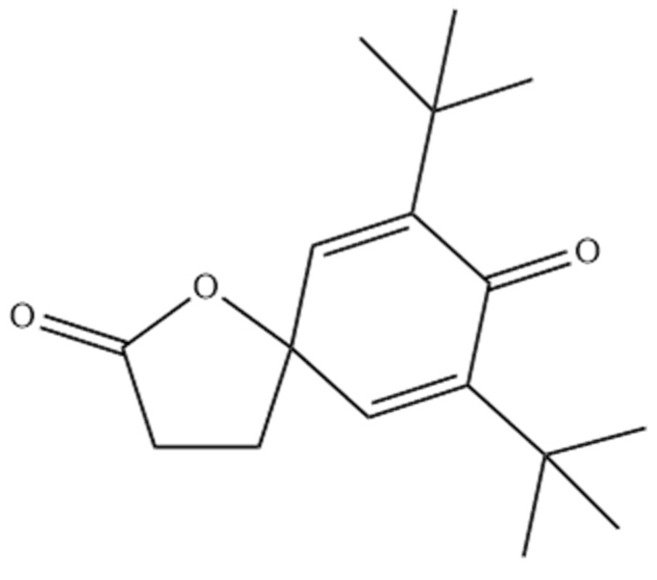
Chemical structure of 7,9-di-tert-butyl-1-oxaspiro[4,5]deca-6,9-diene-2,8-dione.

With respect to compounds coming from the packaging materials, several plasticizers, including phthalates (e.g., diethyl phthalate, butyl octyl phthalate), were identified in almost all samples. Chemicals of phthalate esters (PAEs) can act as endocrine disruptors and lead to adverse effects on organisms even in a low concentration [29]. They can also induce various etiological diseases of humans, such as disorders of the male reproductive tract, breast and testicular cancers, and dysfunction of the neuroendocrine system [30]. Isobenzofuran-1,3-dione, also called phthalic anhydride, was identified in samples BC02, BC05, BC07, and BC10. The most important derivatives of this compound are plasticizers and also polyester resins and dyes [31]. This compound has been classified as high toxicity (Class III), according to Cramer rules. Besides, it can be part of a curing agent system used during the manufacturing of an epoxy resin [32]. Other compounds identified include 2-oxepanone and hexa(methoxymethyl)melamine. The lactone has been reported as a degradation product of polyurethanes and in this study the analysis was carried out by pyrolysis-gas-chromatography/mass spectrometry [33], and hexa(methoxymethyl)melamine is widely employed as a cross-linking agent in coatings [34]. This compound belongs to Class III, according to Cramer rules. A NIAS compound, specifically 7,9-di-tert-butyl-1-oxaspiro[4,5]deca-6,9-diene-2,8-dione (Figure 2), was identified in sample BC05 and it presents high toxicity (Class III). This compound has been reported as a degradation product of the antioxidant Irganox 1010 and has been found in several samples of both plastic and paper packaging and in polyurethane adhesives [35,36]. In plastic materials the analyte was determined in aqueous extracts using the purge and trap method combined with GC-MS [36].

For some compounds, despite their high abundance, identification was not possible with the spectral libraries available, such as the compounds at 28.4 min (*m*/*z* 149, which is the characteristic mass of phthalates compounds), 29.97 min (*m*/*z* 301), 32.45 min (*m*/*z* 345), 35.37 min (*m*/*z* 149), and 35.95 min (*m*/*z* 389). Detailed information about the mass spectra of the unidentified compounds is available in the electronic Supplementary Material.

### 3.4. Can Coatings Analysis via SPME

The compounds detected after the extraction with SPME are summarized in Table 4. Only compounds with appropriate direct matching factors (SI) and reverse search matching (RSI) are included. For those compounds whose identification was not achievable, the most abundant *m*/*z* is specified.

Most of the substances identified are food flavorings authorized in EU. For example, α-terpinene, benzaldehyde, 1-hexanol-2-ethyl, limonene, nonanal, carvone, ethyl-decanoate, and 2-azepanone, among others. Limonene provides a pleasant lemon scent; it is a common compound found in natural products such as resins of plants and in consumer goods such as fruit juices and juices beverages. Additionally, it is used as a raw material to manufacture cardboard or paper [37,38]. In the case of nonanal, it has also been detected in several materials, e.g., paper, polyethylene and polypropylene. It is characterized by a strong odor. The analysis was performed either via gas chromatography-olfactometry-mass spectrometry (GC-O-MS) or by aroma extract dilution analysis with dichloromethane [37,39]. Other common compounds also detected via GC-O-MS were 1-hexanol-2-ethyl, which is produced on a massive scale as a solvent and also as a precursor for the production of plasticizers with a green odor [37]. Benzaldehyde, which was detected in samples BC01 and BC05, has been reported in recycled cardboard [40] as well in adhesives [41]. Moreover, it is a very common natural flavor that might be present in beverages. 2-Azepanone, also known as caprolactam, was detected in all samples analyzed except in sample BC02; besides its use as food flavoring it has a widespread use in food packaging materials and clothing. For example, it was used in coating powders for protective films to block isocyanates [42]. Carcinogenicity studies had considered that 2-azepanone was not carcinogenic under the conditions of the bioassay in F344 rats and B6C3F1 mice [43]. DEP was also extracted with SPME, and this phthalate is the most commonly used plasticizer worldwide in many industrial products, including tools, automotive parts, toothbrushes, food packaging, cosmetics, and insecticides [44]. 2-Butoxyethanol, which was present in all samples, is used as a solvent in coatings formulation [45]. Diphenylmethanone or benzophenone was detected in samples BC01, BC02, and BC03; this substance has been used in polymeric photoinitiators for UV curing coatings [46].

Other compounds like tetramethyl benzenes have been reported in apple juice. The analytes were isolated via SPME using a 50/30 μm divinylbenzene/Carboxen/polydimethylsiloxane (DVB/CAR/PDMS) fiber [47].

Other compounds identified in several samples were propylene glycol and 2,2-dimethyl-1,3-propanediol, also known as neopentyl glycol, which are commonly employed in the manufacturing of polyurethanes [48,49].

2-Phenoxyethanol was only identified in sample BC04. This compound has been reported in TritanTM copolyester, a potential substitute of polycarbonate. The compound was determined in aqueous extracts purified with solid-phase extraction (SPE) and then analyzed via GC-MS [50]. Degradation products of the antioxidants Irgafos 168, Irganox 1076, or Irganox 1010, specifically 2,4-ditertbutylphenol and 2, 6-di-tert-butyl-1,4-benzoquinone, were identified in various samples [51]. 2, 6-Di-tert-butyl-1,4-benzoquinone presents intermediate toxicity (Class II). These compounds have been reported as NIAS. Only two of the identified compounds, 2-azepanone and diphenylmethanone, belong to Cramer Class III.

## 4. Conclusions

A wide variety of volatile and semi-volatile low molecular weight compounds were identified in polymeric coatings for metal beverage cans via solvent extraction and SPME followed by GC-MS. Fifty-six compounds were detected when using HS-SPME-GC-MS and 35 when the extraction solvent was applied. Esters were the predominant compounds determined via solvent extraction, whereas aromatic compounds and aldehydes were the most abundant compounds determined via SPME. From our results, the SPME method seems to be a more suitable identification technique, in terms of the number of compounds detected, and in general good library matches were obtained compared to the other technique. Besides, it is an eco-friendly and solvent-free extraction technique.

## Figures and Tables

**Table 1:** Sample descriptions.

Code	Beverage	Polymeric Coating	Thickness (µm)
BC01	Traditional Beer	Lat. Ext.: PU	Lateral: 114.5 Lid: 313.0
		Lat. Int.: Phx	
		Lid Int.: Phx	
		Lid Ext.: Phx	
BC02	Vodka mixed drink	Lat. Ext.: PU	Lateral: 109.0 Lid: 218.0
		Lat. Int.: Phx	
		Lid Int.: Epx	
		Lid Ext.: Epx	
BC03	Mixed lemon flavour	Lat. Ext.: PU	Lateral: 104.0 Lid: 218.0
		Lat. Int.: Phx	
		Lid Int.: Phx	
		Lid Ext.: Phx	
BC04	Energy Drink Zero	Lat. Ext.: PU	Lateral: 112.0 Lid: 264.0
		Lat. Int.: Phx	
		Lid Int.: Epx	
		Lid Ext.: Epx	
BC05	Star Wars Space Punch	Lat. Ext.: PP	Lateral: 114.0 Lid: 331.0
		Lat. Int.: Acrylic	
		Lid Int.: Polyester	
		Lid Ext.: Phx	
BC06	Green cola	Lat. Ext.: PU	Lateral: 115.0 Lid: 234.0
		Lat. Int.: Phx	
		Lid Int.: Phx	
		Lid Ext.: Phx	
BC07	Tonic original	Lat. Ext.: PU	Lateral: 111.0 Lid: 258.0
		Lat. Int.: Phx	
		Lid Int.: Epx	
		Lid Ext.: Epx	
BC08	Tonic water original	Lat. Ext.: PU	Lateral: 113.0 Lid: 230.0
		Lat. Int.: Phx	
		Lid Int.: Epx	
		Lid Ext.: Epx	
BC09	Premium tonic water	Lat. Ext.: PU	Lateral: 123.0 Lid: 226.0
		Lat. Int.: Acrylic	
		Lid Int.: Polyester	
		Lid Ext.: Phx	
BC10	Natural mineral water drink	Lat. Ext.: PU	Lateral: 103.0 Lid: 248.0
		Lat. Int.: Phx	
		Lid Int.: Epx	
		Lid Ext.: Epx	

Epx: Epoxy resin; Ext.: external; Int: Internal; Lat: Lateral; Phx: Phenoxy resin; PP: Polypropylene; PU: Polyurethane.

**Table 2:** Comparison of the most abundant detected compounds in sample BC04 using different extraction solvents.

Tr/min	Compound	CAS	Fl No.	MeOH	ACN	Hex	Hex: EtOH (3:1 *v*/*v*)
10.56	Benzoic acid methyl ester	93-58-3	09.725	x			
11.01	Caprylic acid methyl ester	111-11-5	09.117	x			
12.97	Adipic acid methyl ester	627-93-0		x			
17.17	Lauric acid methyl ester	111-82-0	09.101	x			
17.55	Unknown compound (*m*/*z* 129)			x			
18.07	Diethyl phthalate *	84-66-2		x		x	x
19.92	Ester			x			
20.25	Unknown compound (*m*/*z* 56)			x			
21.71	Thiophene			x			
25.07	Unknown compound (*m*/*z* 151)			x			
25.56	Adipate structure			x	x	x	x
25.65	Adipate structure			x	x	x	x
30.43	Unknown compound			x	x	x	x

* Substances confirmed with a standard solution.

**Table 3:** Compounds identified via GC-MS analysis after extraction with MeOH as a solvent.

Tr/min	Compound	CAS	Fl No.	SI	RSI	Sample(s)	TC
10.56	Benzoic acid methyl ester	93-58-3	09.725	745	857	BC04	I
11.01	Caprylic acid methyl ester	111-11-5	09.117	701	789	BC04	I
11.37	2-Oxepanone	502-44-3		729	862	BC06, BC07	I
12.34	α-Terpineol *	98-55-5	02.014	902	936	9	III
12.97	Adipic acid methyl ester	627-93-0		794	867	BC01, BC03, BC04, BC06–BC08	I
14.2	Isobenzofuran-1,3-dione	85-44-9		841	922	BC02, BC05, BC07, BC10	III
15.58	Unknown diol					BC05	
16.93	(+)-Ledene	21747-46-6		893	927	BC09	I
17.17	Lauric acid methyl ester	111-82-0	09.101	855	878	BC01–BC10	I
17.55	Ester structure (*m*/*z* 129)					BC01–BC10	
18.08	Diethyl phthalate *	84-66-2		929	938	BC01–BC05, BC09, BC10	I
18.37	Unknown compound (*m*/*z* 107, 163)					BC09	
19.28	Dodecalactone	2305-05-7	10.019	855	894	BC05	II
19.92	Ester structure (*m*/*z* 129)					BC01–BC10	
20.29	Unknown compound (*m*/*z* 56, 111)					BC08	
20.72	Butyl octyl phthalate	84-78-6		714	758	BC02	I
20.99	Ketone structure					BC05, BC07	
21.53	Unknown compound *m*/*z* (45, 109)					BC05	
21.73	2-Isobutyl-5-propylthiophene	4861-63-6				BC01–BC06, BC08–BC10	III
21.99	7,9-Di-tert-butyl-1-oxaspiro[4,5]deca-6,9-diene-2,8-dione	82304-66-3				BC05	III
22.33 and 23.3	Unknown compound (Phthalate structure *m*/*z*: 149)					BC02, BC05, BC07	
23.81	Unknown compound (*m*/*z* 151)					BC06–BC08, BC10	
25.08	Unknown compound (*m*/*z* 151)					BC01, BC02, BC04, BC08–BC10	
25.56	Unknown compound (*m*/*z* 129, 111)					BC01–BC10	
25.65	Unknown compound (*m*/*z* 129, 111)					BC01–BC010	
26.53	Unknown compound (*m*/*z* 163)					BC08, BC10	
27.19	α-Methyl-δ-oxo-2-phenyl-1,3-dioxolane-2-heptanenitrile	58422-90-5		782	940	BC02, BC05, BC07, BC09, BC10	III
27.42	Hexa(methoxymethyl)melamine	68002-20-0		857	874	BC01- BC03, BC05, BC08, BC10	III
27.87	Unknown compound (*m*/*z* 143, 111)					BC08	
28.4	Unknown compound (Phthalate structure *m*/*z* 149)					BC02, BC05, BC07, BC09, BC10	
29.96	Unknown compound (*m*/*z* 301)					BC02, BC05, BC09, BC10	
30.46	Unknown compound (*m*/*z* 69, 81)					BC08	
32.45	Unknown compound (*m*/*z* 345)					BC02, 5 BC0, BC09, BC10	
35.36	Unknown compound (Phthalate structure *m*/*z*: 149)					BC02, BC05, BC010	
35.95	Unknown compound (*m*/*z* 389)					BC02, BC05, BC09	

* Substances confirmed with a standard solution.

**Table 4:** Compounds identified via SPME GC-MS analysis.

Tr/min	Compound	CAS	Fl No.	SI	RSI	Sample(s)	TC
9.61	Propylene glycol	57-55-6		571	818	BC04	I
13.72	2-Butoxyethanol *	111-76-2	02.242	866	925	BC01–BC10	I
15.37	α-Terpinene	99-86-5	01.019	586	754	BC09	I
15.61	Benzaldehyde	100-52-7	05.013	631	865	BC01, BC05	I
15.85	2,2-Dimethyl-1,3-Propanediol *	126-30-7		821	892	BC01, BC02, BC03, BC04, BC08	I
16.11	Octanal *	124-13-0	05.009	505	701	BC04	I
16.28	1,2,3,4-Tetramethyl benzene	488-23-3		849	879	BC09	I
16.43	Limonene *	5989-27-5	01.045	916	924	BC02, BC03, BC05–BC08	I
16.5	p-Cymene	99-87-6	01.002	909	926	BC02, BC03, BC06, BC09	I
16.69	1-Hexanol-2-ethyl	104-76-7	02.082	809	913	BC05, BC06, BC08	I
17.05	g-Terpinene	99-85-4	01.020	882	894	BC02, BC09	I
17.68	Terpinolene	586-62-9	01.005	795	864	BC02	I
18.07	Benzene structure					BC02, BC05, BC07, BC09	
18.45	Nonanal *	124-19-6	05.025	701	857	BC01–BC04, BC06	I
19.02	Unknown compound (*m*/*z* 79, 121; cyclohexenol structure)					BC05	
19.35 and 20.51	Unknown compound (*m*/*z* 134; cyclohexanol structure)					BC05	
19.39	Benzenemethanol	60-12-8	02.019	842	896	BC01, BC03	I
19.92	Ethyl octanoate	106-32-1	09.111	913	938	BC01–BC03	I
20.35	Octanoic acid	124-07-2	08.010	763	928	BC01, BC02	I
20.61	Decanal	112-31-2	05.010	853	924	BC02, BC04, BC06–BC08	I
20.74	Unknown compound (*m*/*z* 70, 119; ester structure)					BC05	
20.92	Benzoic acid *	65-85-0	08.021	917	931	BC04	I
21.43	Unknown compound (*m*/*z* 109, 71; cyclohexanol structure)					BC05	
21.5	Unknown compound (*m*/*z* 135, 79, 107)					BC05, BC07	
21.67	2-Phenoxyethanol *	122-99-6		696	965	BC04	I
21.68	2-Phenethyl acetate	103-45-7	09.031	822	911	BC01	I
21.76	Carvone	99-49-0	07.012	860	914	BC05, BC07, BC09	II
22.15	Nonanoic acid	112-05-0	08.029	806	915	BC01, BC04	I
22.36	Unknown compound (*m*/*z* 73)					BC03	
22.47	Undecanal	112-44-7	05.034	633	824	BC04	I
22.98	2-Azepanone *	105-60-2	16.052	883	889	BC01, BC03–BC10	III
23.57	Ethyl-decanoate	110-38-3	09.059	902	935	BC01–BC03, BC05	I
23.92	Decanoic acid	334-48-5	08.011	671	775	BC04	I
24.25	Dodecanal	112-54-9	05.011	606	872	BC02, BC06, BC0 7	I
24.68	2-Methylaminobenzoic acid	85-91-6	09.781	676	933	BC04	I
25.05	6,10-Dimethyl-5,9-undecadien-2-one	3796-70-1	07.123	688	773	BC02	I
25.39	Benzaldehyde-4-hydroxy-3-methoxy *	121-33-5	05.018	830	904	BC04	I
25.51	Napthalene structure			792	833	BC09	
25.54	2, 6-Di-tert-butyl-1,4-benzoquinone (2,6-DTBQ) *	719-22-2		729	804	BC06	II
25.77	1,3-Diacetylbenzene	6781-42-6		818	875	BC01	I
26.34	2,4-Ditertbutylphenol	97-76-4				BC03, BC06–BC08, BC10	I
26.40	Decalactone-g	706-14-9	10.017	887	912	BC05, BC09	II
26.49	Unknown compound (*m*/*z* 43, 163,120; phenol structure)					BC01	
26.78	Dodecanoate-ethyl	106-33-2	09.099	879	935	BC01	I
27.39	Unknown compound (*m*/*z* 129, 111; ester structure)					BC03	
27.75	Diethyl phthalate *	84-66-2		922	930	BC01–BC10	I
28.03	Undecalactone-g	104-67-6	10.002	755	849	BC05, BC07	II
28.28	Unknown compound (*m*/*z* 213,109)					BC05	
28.32	Alcohol					BC01, BC04	
28.60	Diphenylmethanone *	119-61-9	07.032	717	941	BC01- BC03	III
29.56	Lactone structure					BC05	
29.69	Unknown compound (*m*/*z* 81, 99; methanone structure)					BC01, BC07	
30.07	Tetradecanoate	110-27-0	09.105	619	848	BC01, BC04	I
30.65	Unknown compound (*m*/*z* 219,191)					BC08	
30.85	Unknown compound (*m*/*z* 69)					BC08	
31.85	Phthalate structure (*m*/*z* 149)					BC04, BC08	

* Substances confirmed with a standard solution.

## Data Availability

The data presented in this study are available in [Identification of Volatile and Semi-Volatile Compounds in Polymeric Coatings Used in Metal Cans by GC-MS and SPME and Supplementary Material Identification of volatile and semi-volatile compounds in polymeric coatings used in metal cans by GC-MS and SPME].

## Supplementary Materials

The following are available online at https://www.mdpi.com/article/10.3390/ma14133704/s1, Figure S1–S30: Mass spectra of unknown compounds.
