# Supplementary material for: Identification of Volatile and Semi-Volatile Compounds in Polymeric Coatings Used in Metal Cans by GC-MS and SPME
Source: Materials (Basel). 2021 Jul 2;14(13):3704. doi: 10.3390/ma14133704 (PMC8269810; doi:10.3390/ma14133704)
Supplement: Supplementary file 1 [file materials-14-03704-s001.zip › materials-1265905-supplementary.pdf]

Materials

Electronic Supplementary Material

Identification of volatile and semi-volatile compounds in polymeric coatings used in metal cans by GC-MS and SPME

Patricia Vázquez-Loureiro, Antía Lestido-Cardama, Raquel Sendón, Julia López-Hernández, Perfecto Paseiro-Losada and Ana Rodríguez-Bernaldo de Quirós

Mass spectra of unknown compounds

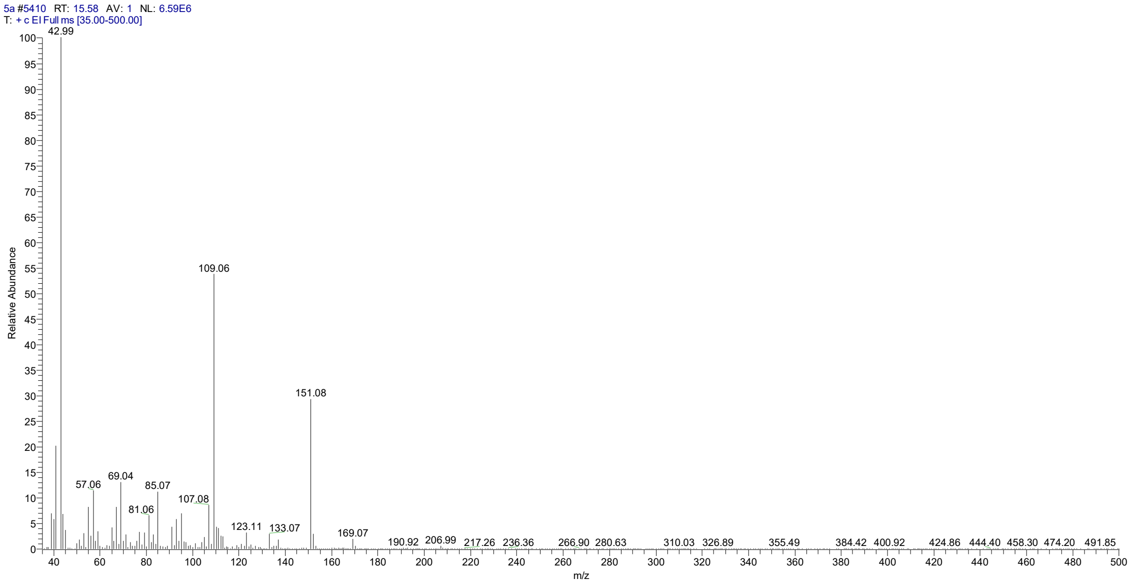

Figure S1.- Solvent extraction GC-MS analysis. Mass spectra of peak at retention time 15.58 minutes.

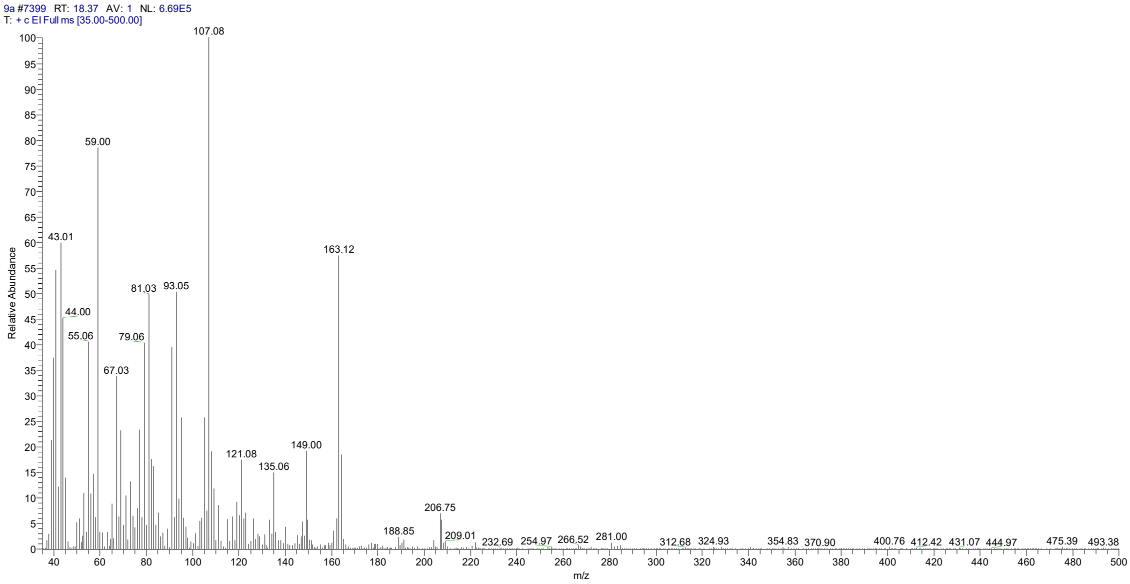

Figure S2.- Solvent extraction GC-MS analysis. Mass spectra of peak at retention time 18.37 minutes.

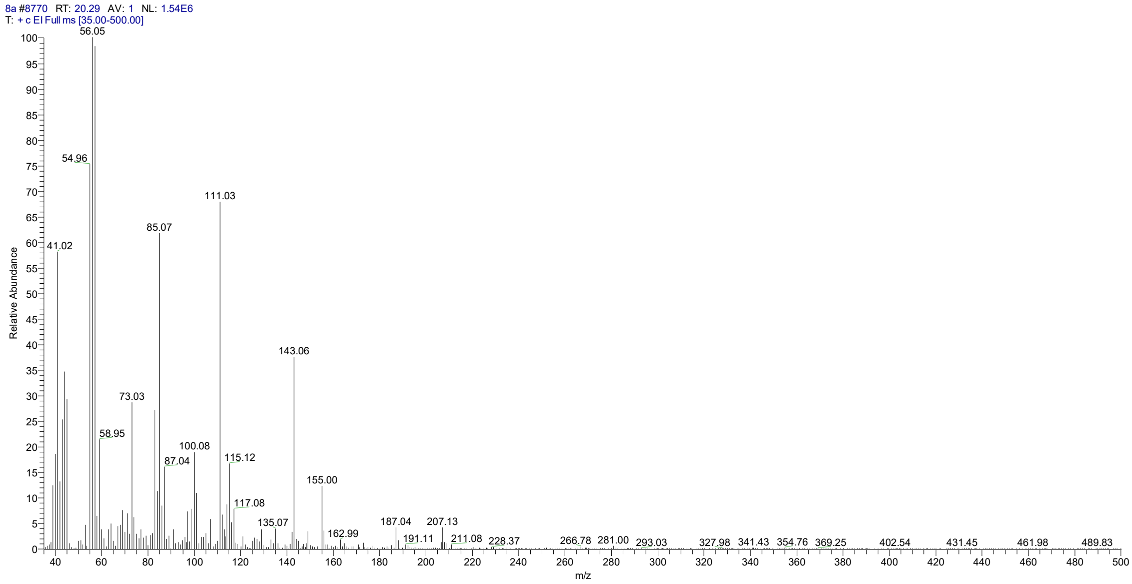

Figure S3.- Solvent extraction GC-MS analysis. Mass spectra of peak at retention time 20.29 minutes.

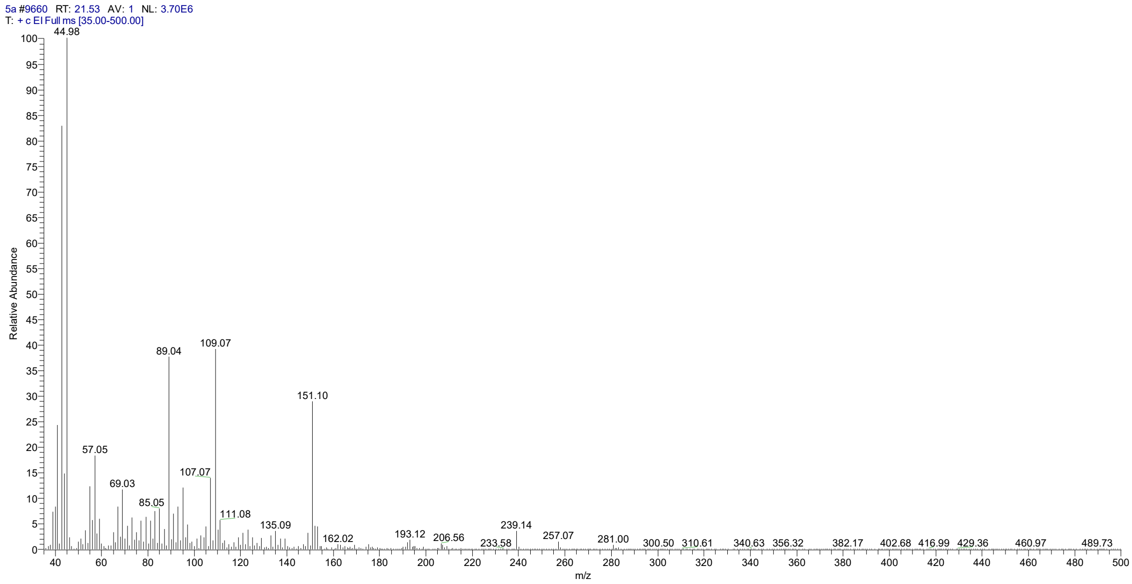

Figure S4.- Solvent extraction GC-MS analysis. Mass spectra of peak at retention time 21.53 minutes.

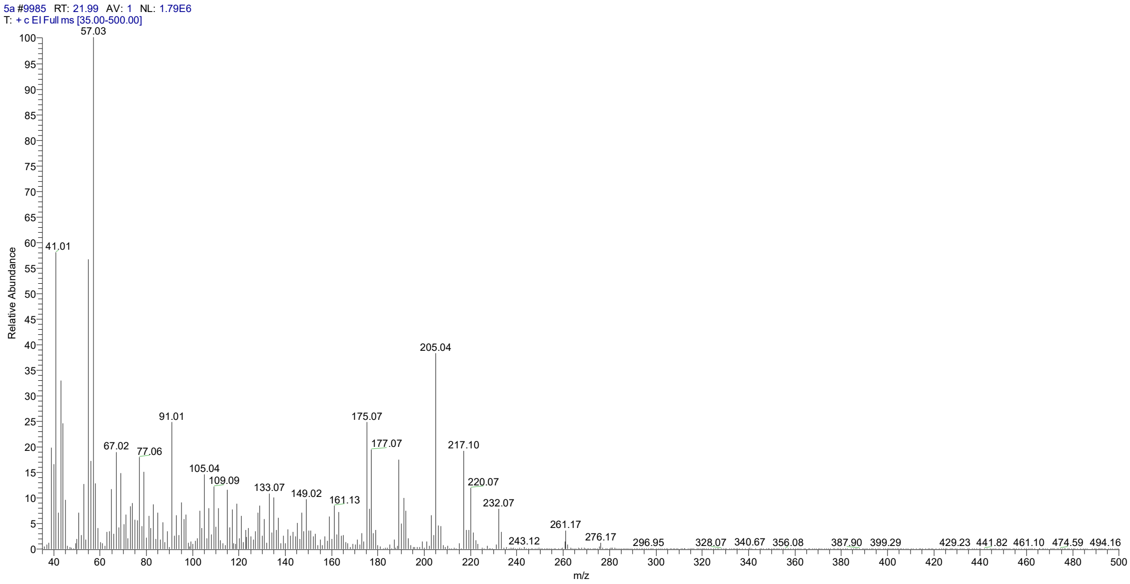

Figure S5.- Solvent extraction GC-MS analysis. Mass spectra of peak at retention time 21.99 minutes.

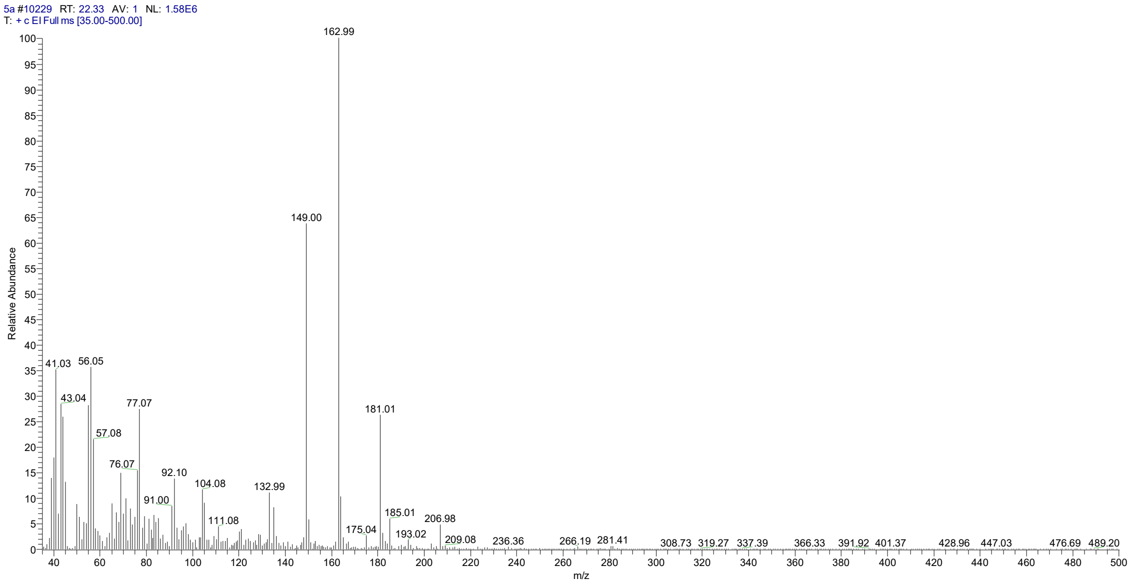

Figure S6.- Solvent extraction GC-MS analysis. Mass spectra of peak at retention time 22.33 minutes.

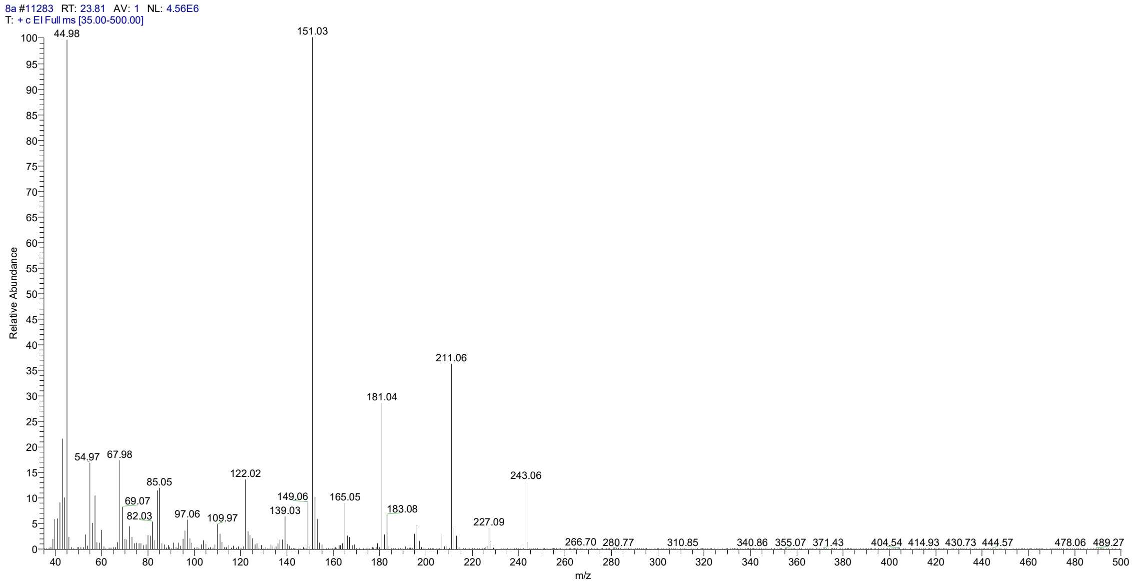

Figure S7.- Solvent extraction GC-MS analysis. Mass spectra of peak at retention time 23.81 minutes.

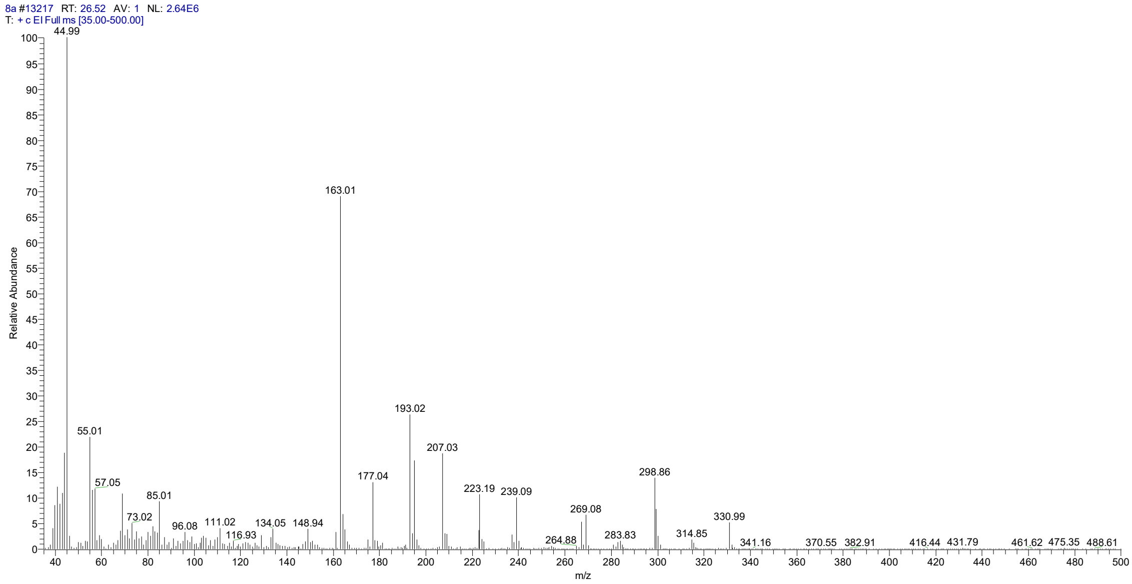

Figure S8.- Solvent extraction GC-MS analysis. Mass spectra of peak at retention time 26.52 minutes.

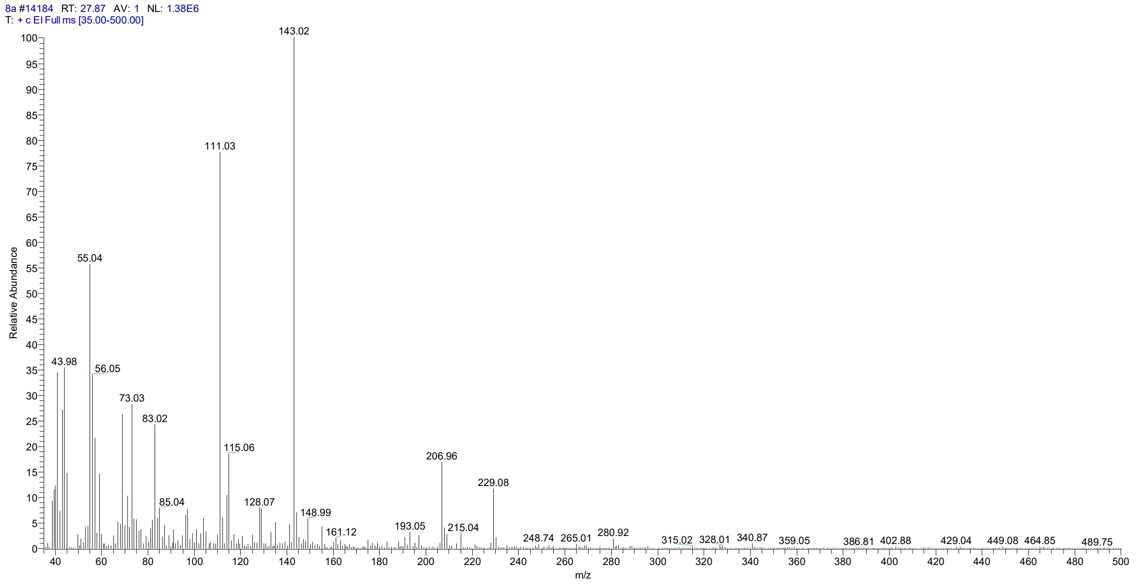

Figure S9.- Solvent extraction GC-MS analysis. Mass spectra of peak at retention time 27.87 minutes.

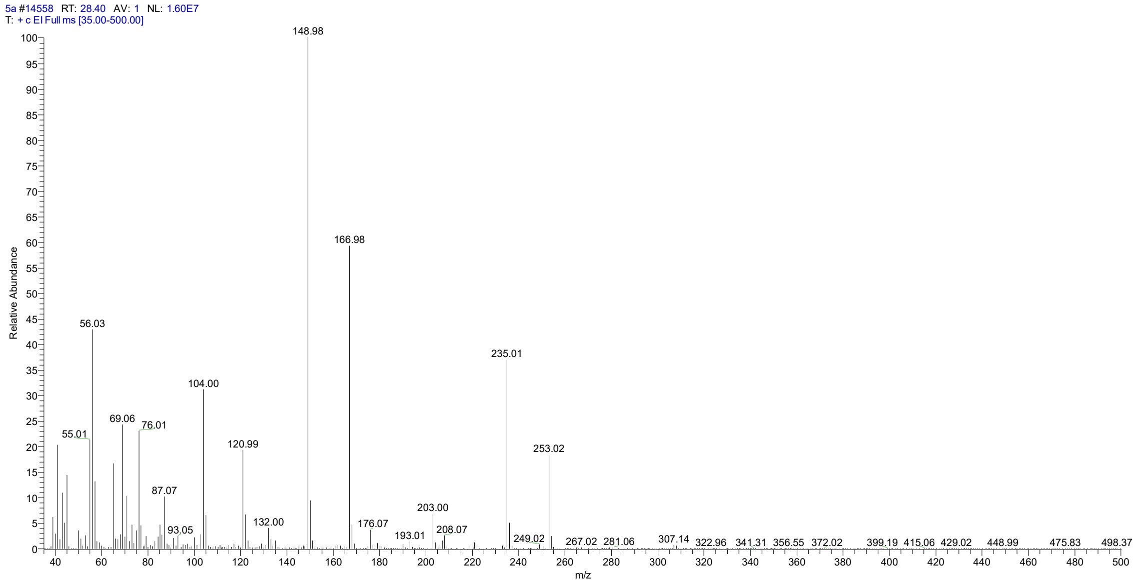

Figure S10.- Solvent extraction GC-MS analysis. Mass spectra of peak at retention time 28.40 minutes.

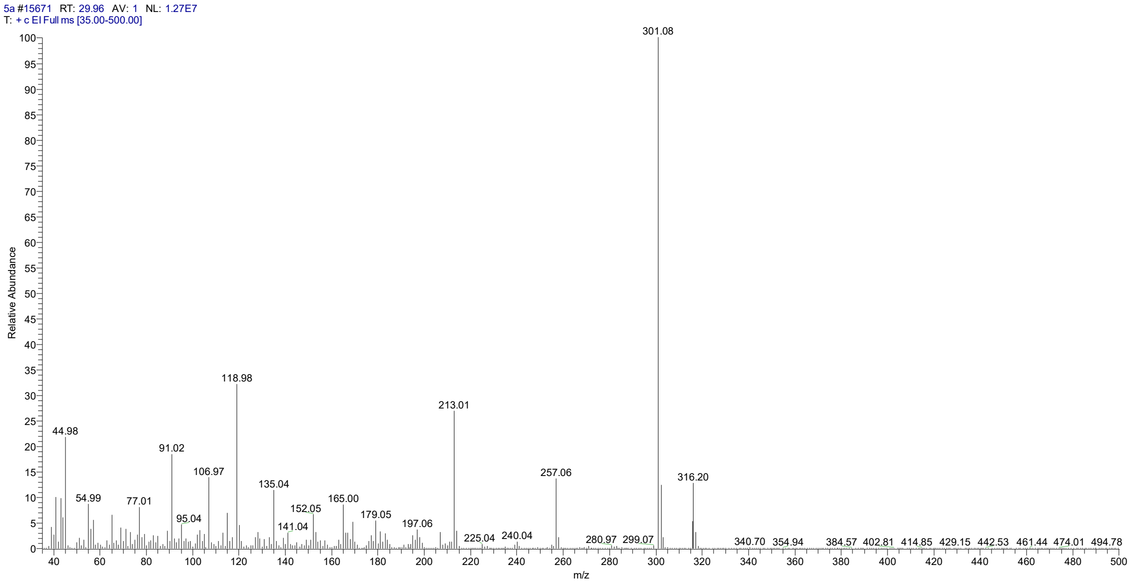

Figure S11.- Solvent extraction GC-MS analysis. Mass spectra of peak at retention time 29.96 minutes.

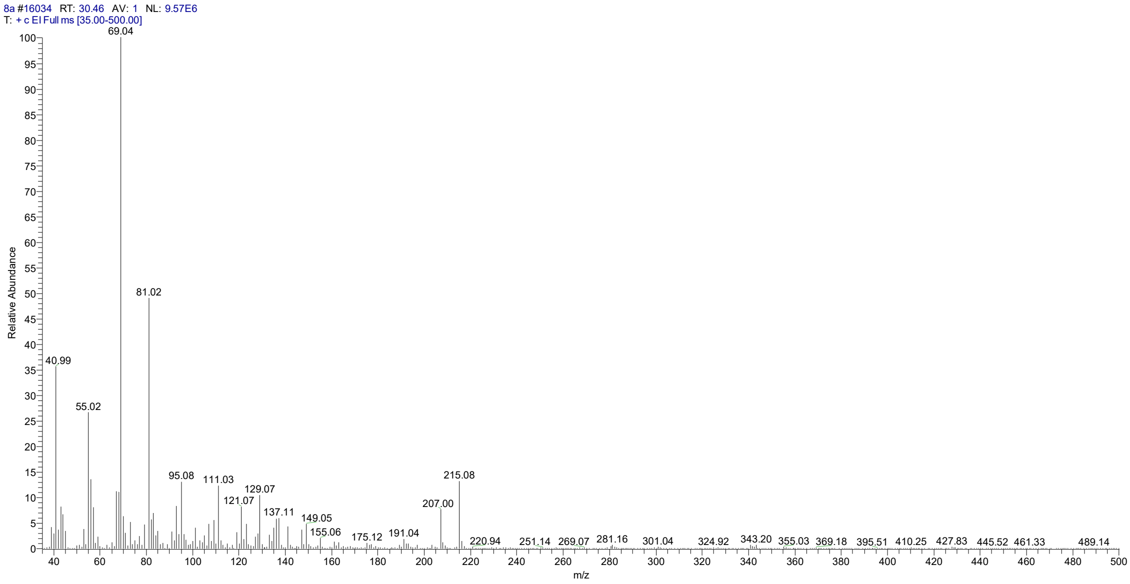

Figure S12.- Solvent extraction GC-MS analysis. Mass spectra of peak at retention time 30.46 minutes.

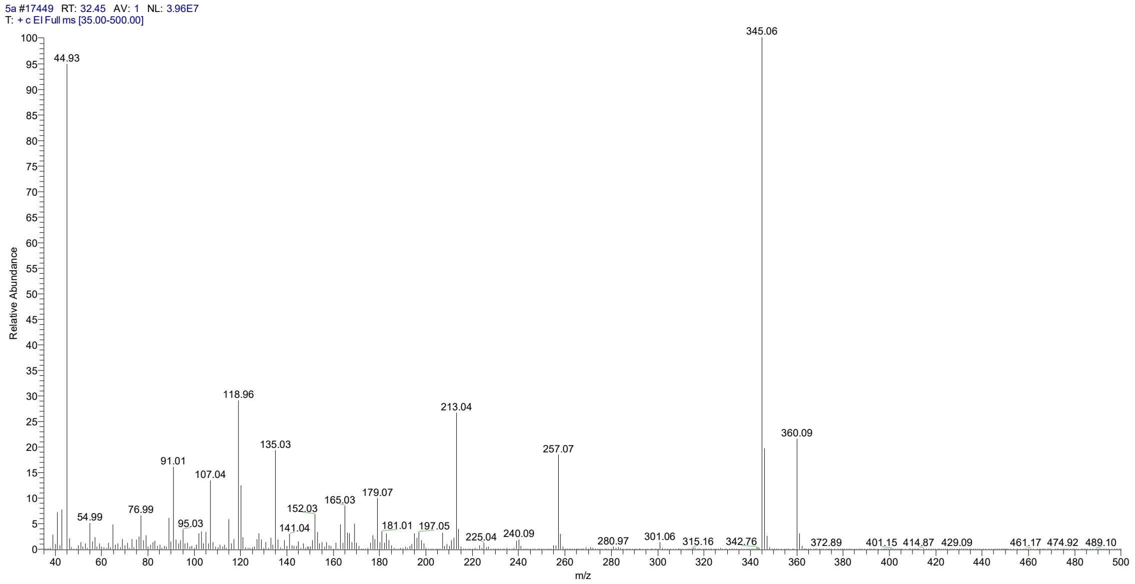

Figure S13.- Solvent extraction GC-MS analysis. Mass spectra of peak at retention time 32.45 minutes**.**

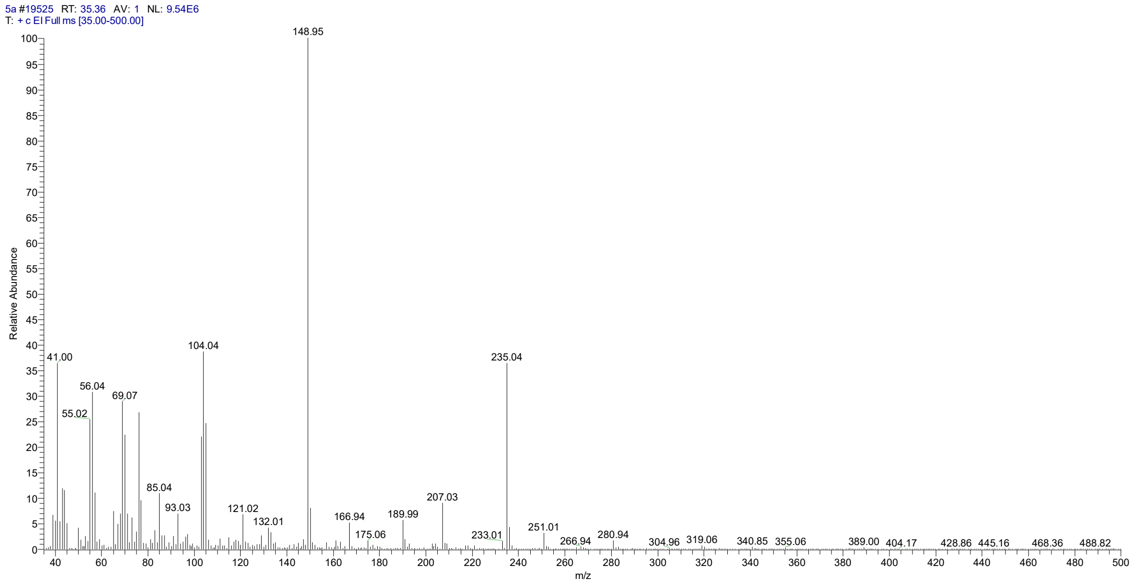

Figure S14.- Solvent extraction GC-MS analysis. Mass spectra of peak at retention time 35.36 minutes.

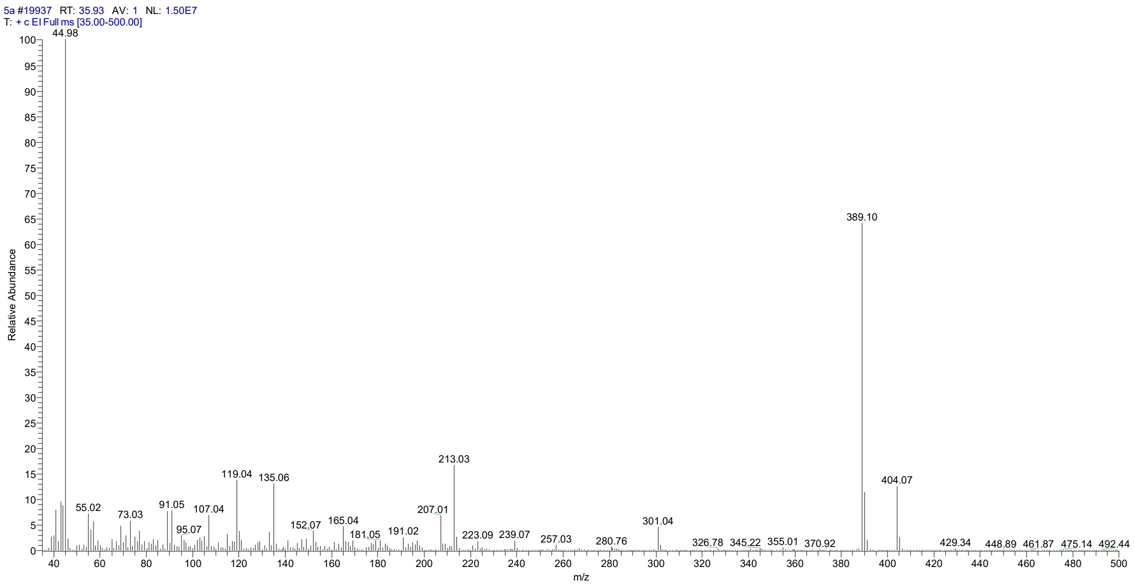

Figure S15.- Solvent extraction GC-MS analysis. Mass spectra of peak at retention time 35.93 minutes**.**

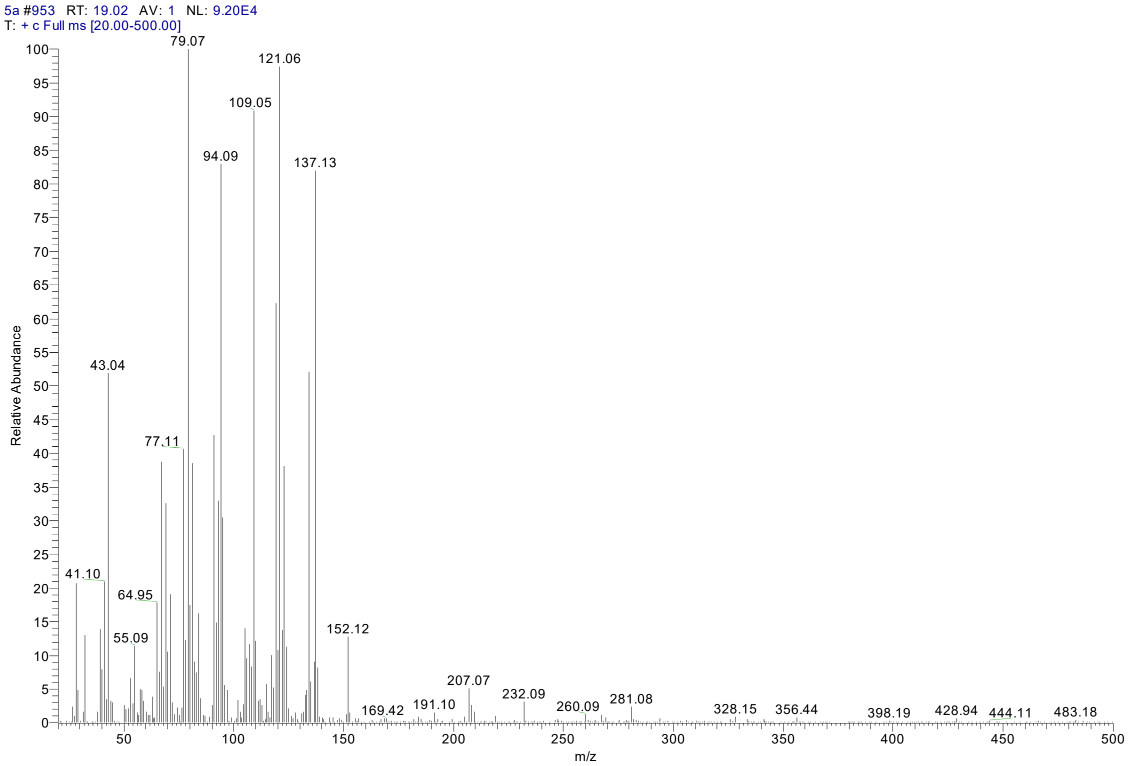

Figure S16.- SPME sampling GC-MS analysis**.** Mass spectra of peak at retention time 19.02 minutes.

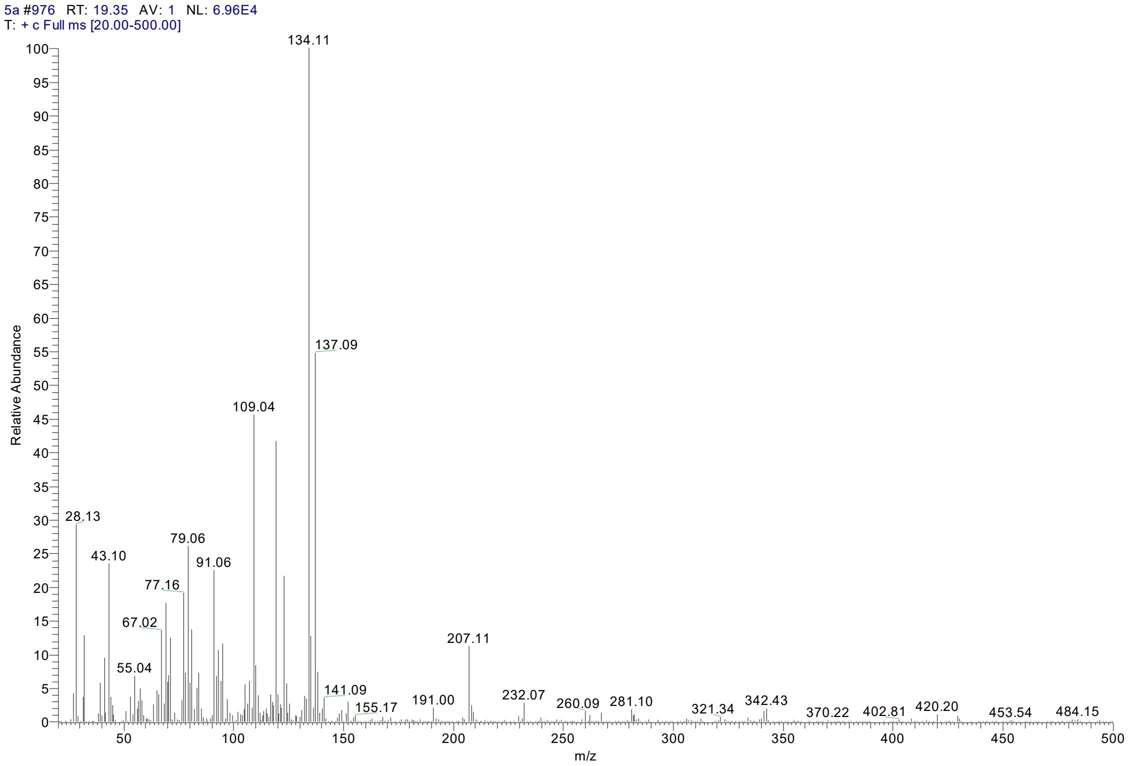

Figure S17.- SPME sampling GC-MS analysis**.** Mass spectra of peak at retention time 19.35 minutes.

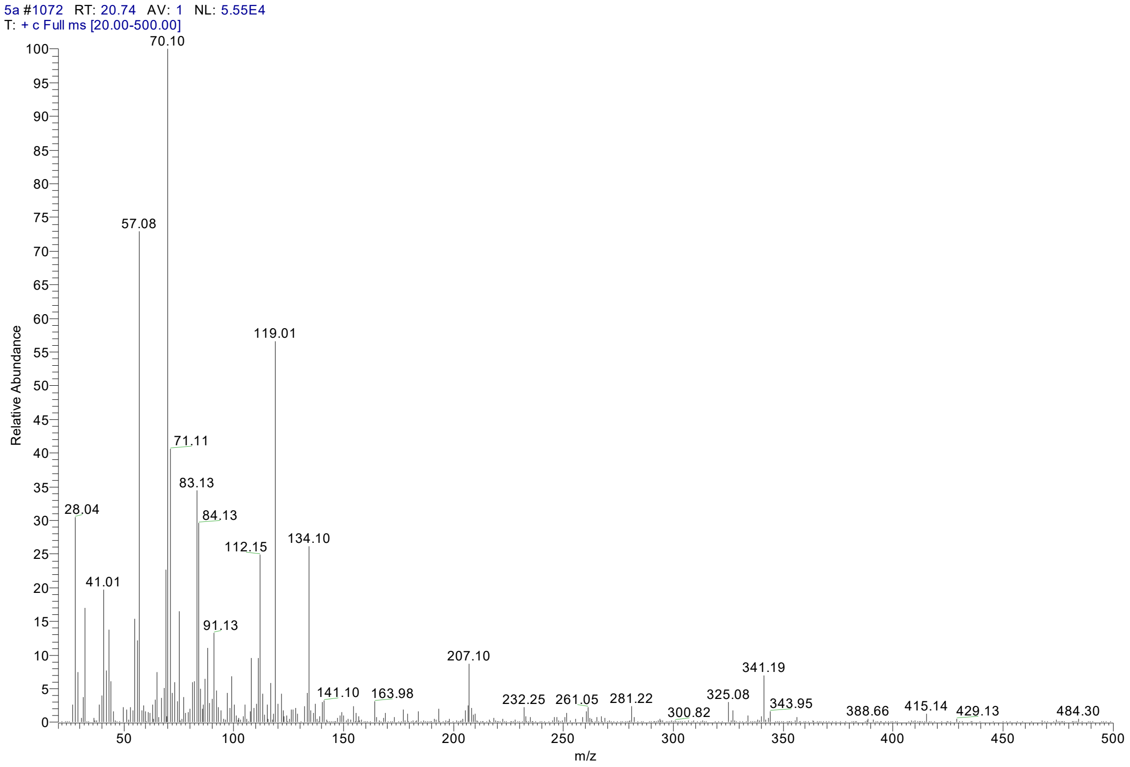

Figure S18.- SPME sampling GC-MS analysis**.** Mass spectra of peak at retention time 20.74 minutes

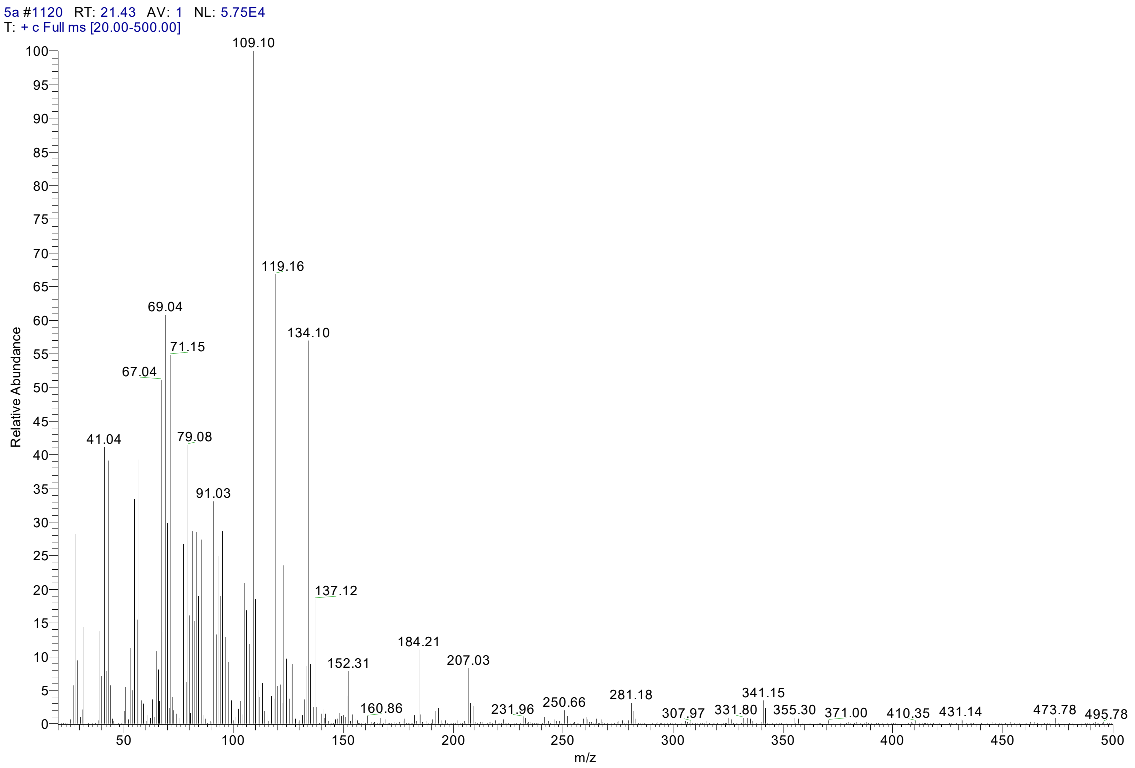

Figure S19.- SPME sampling GC-MS analysis**.** Mass spectra of peak at retention time 21.43 minutes

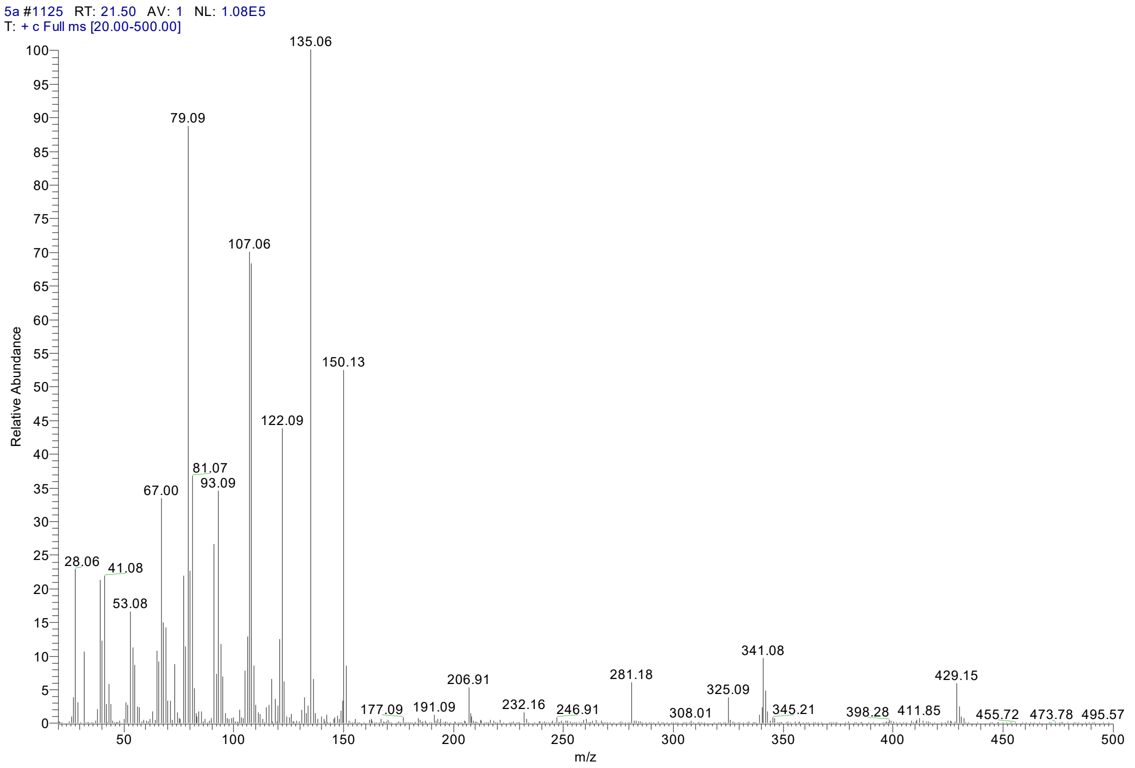

Figure S20.- SPME sampling GC-MS analysis**.** Mass spectra of peak at retention time 21.50 minutes

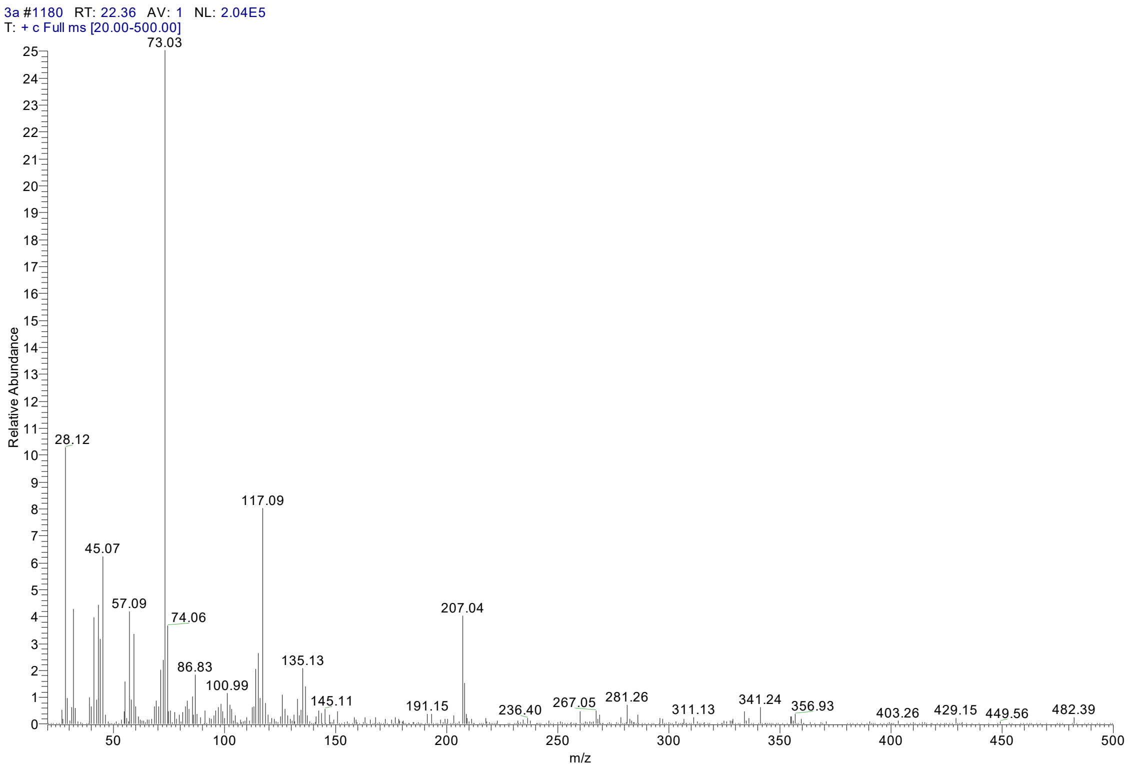

Figure S21.- SPME sampling GC-MS analysis**.** Mass spectra of peak at retention time 22.36 minutes

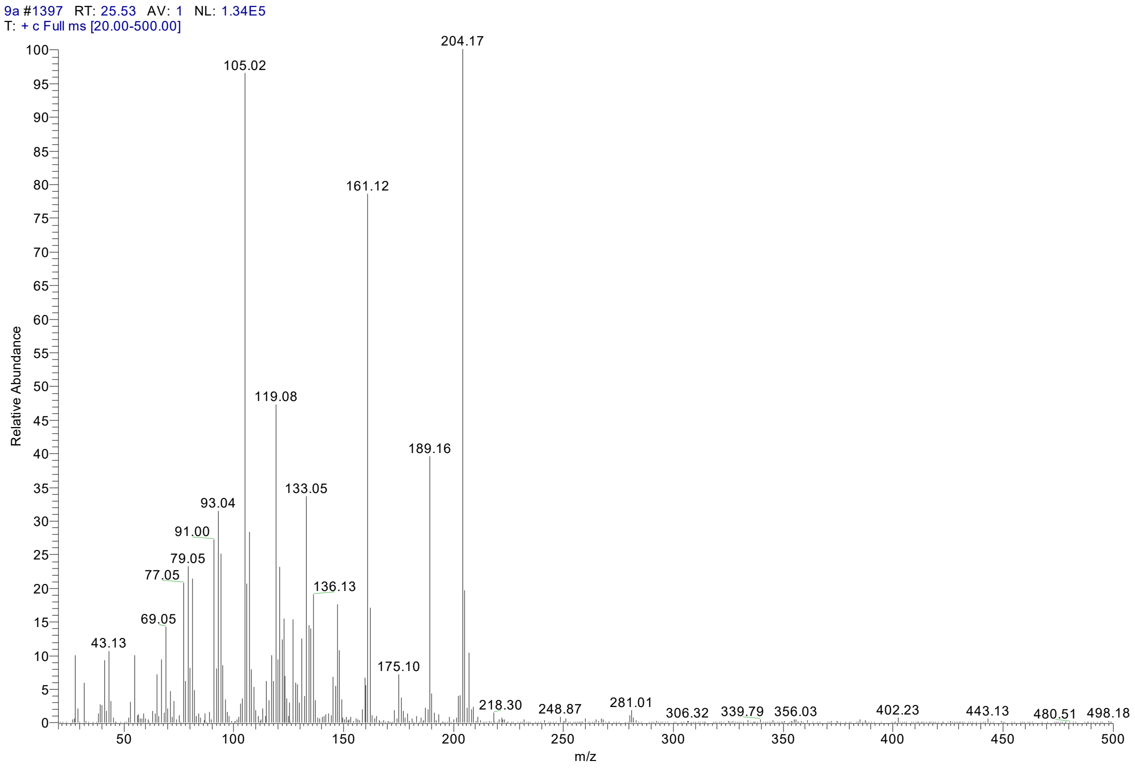

Figure S22.- SPME sampling GC-MS analysis**.** Mass spectra of peak at retention time 25.53 minutes

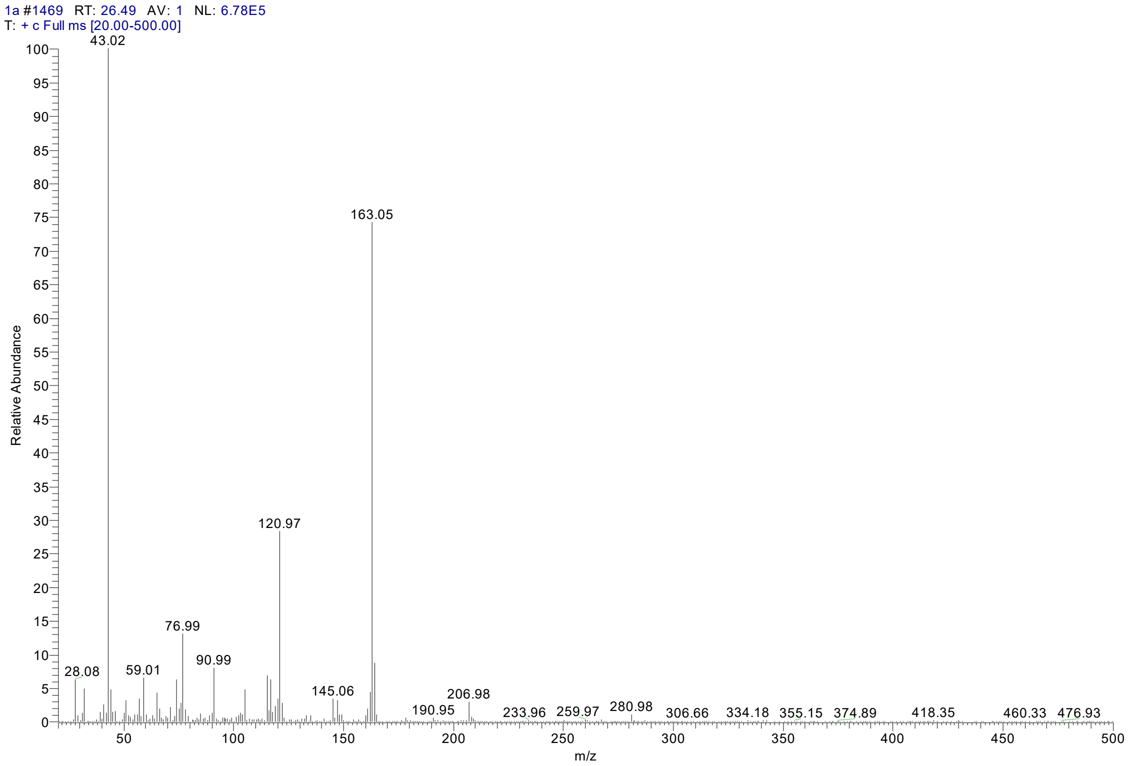

Figure S23.- SPME sampling GC-MS analysis**.** Mass spectra of peak at retention time 26.49 minutes.

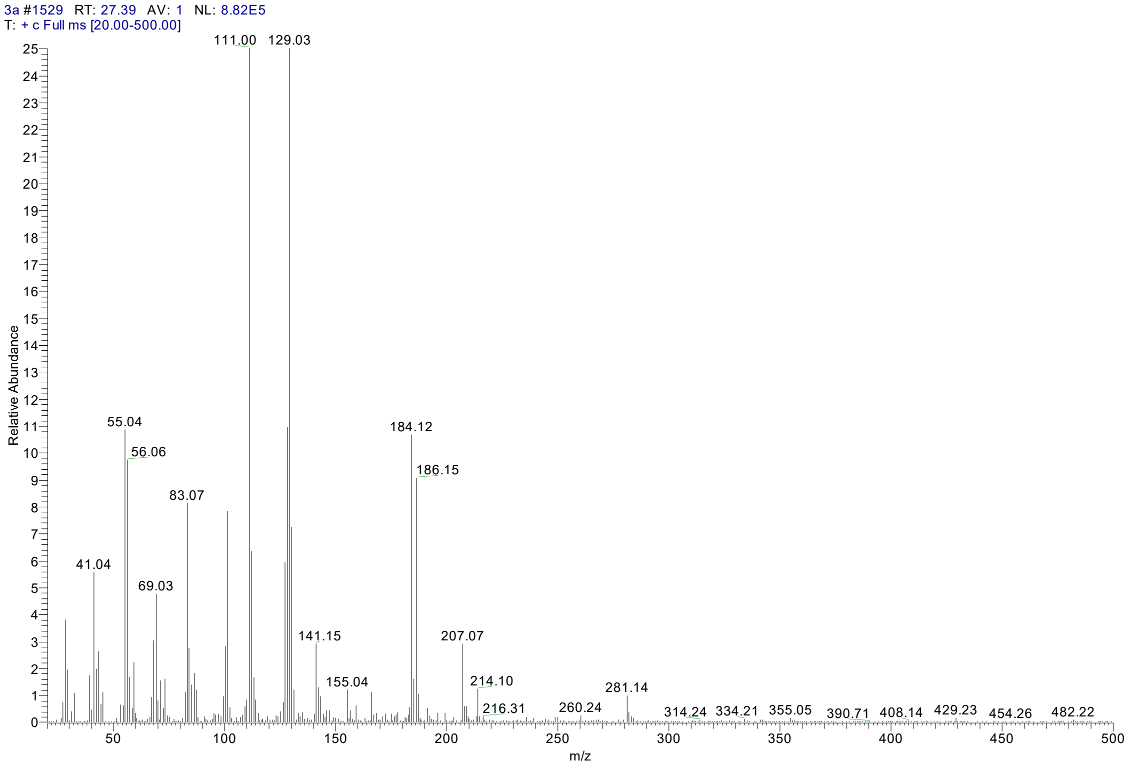

Figure S24.- SPME sampling GC-MS analysis**.** Mass spectra of peak at retention time 27.39 minutes.

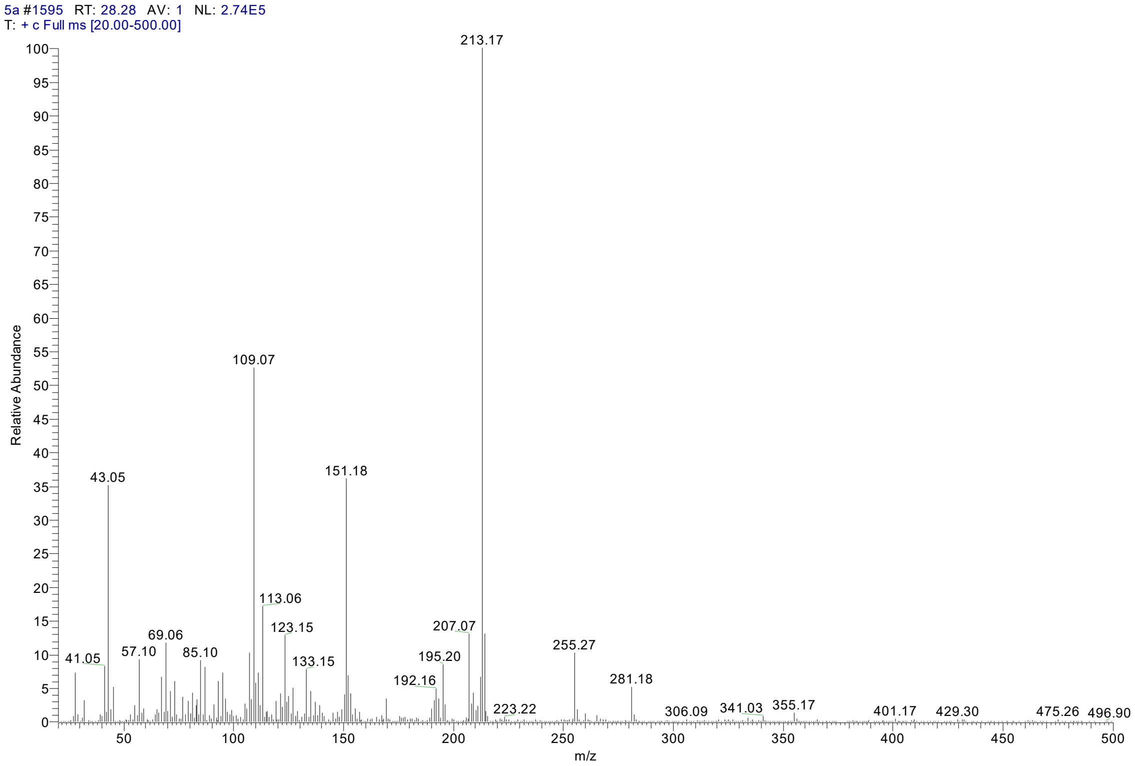

Figure S25.- SPME sampling GC-MS analysis**.** Mass spectra of peak at retention time 28.28 minutes.

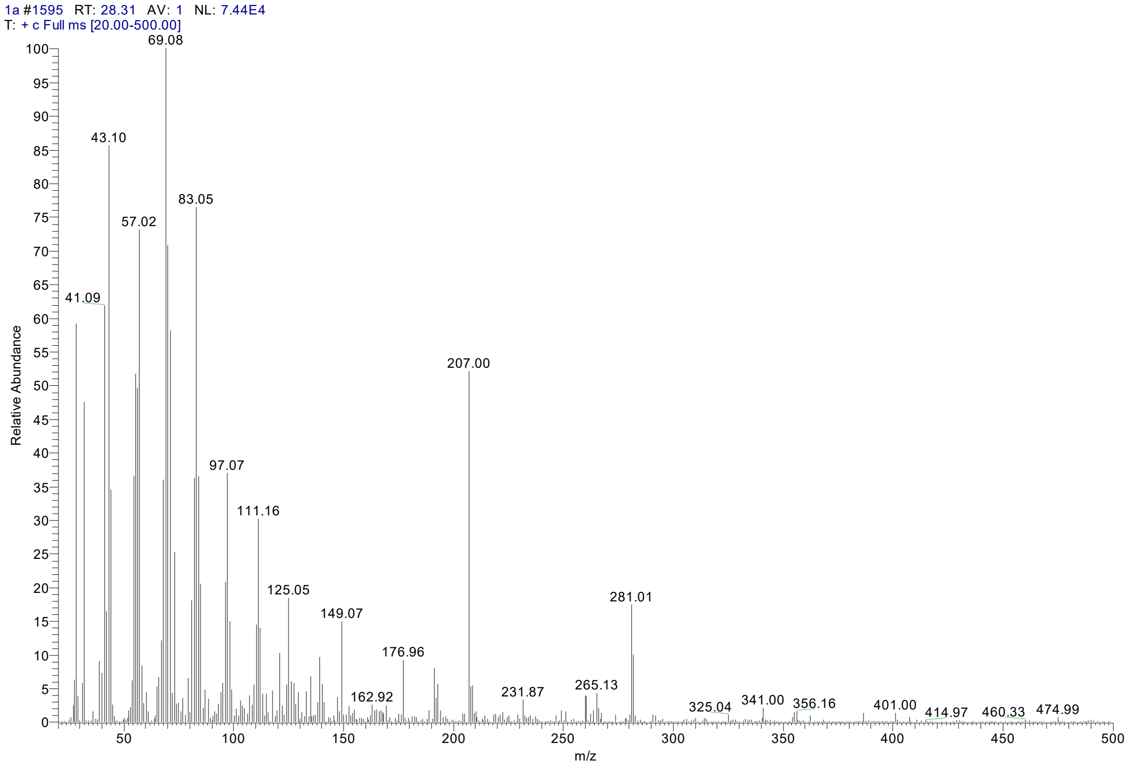

Figure S26.- SPME sampling GC-MS analysis**.** Mass spectra of peak at retention time 28.31 minutes.

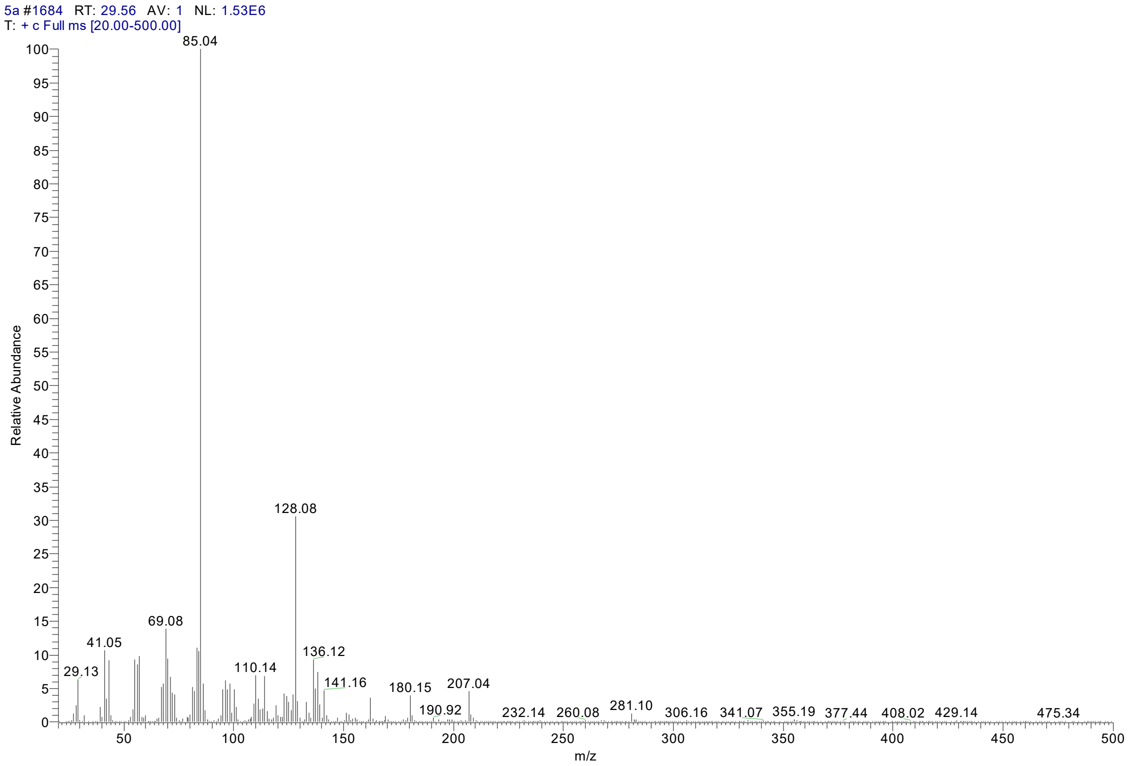

Figure S27.- SPME sampling GC-MS analysis**.** Mass spectra of peak at retention time 29.56 minutes.

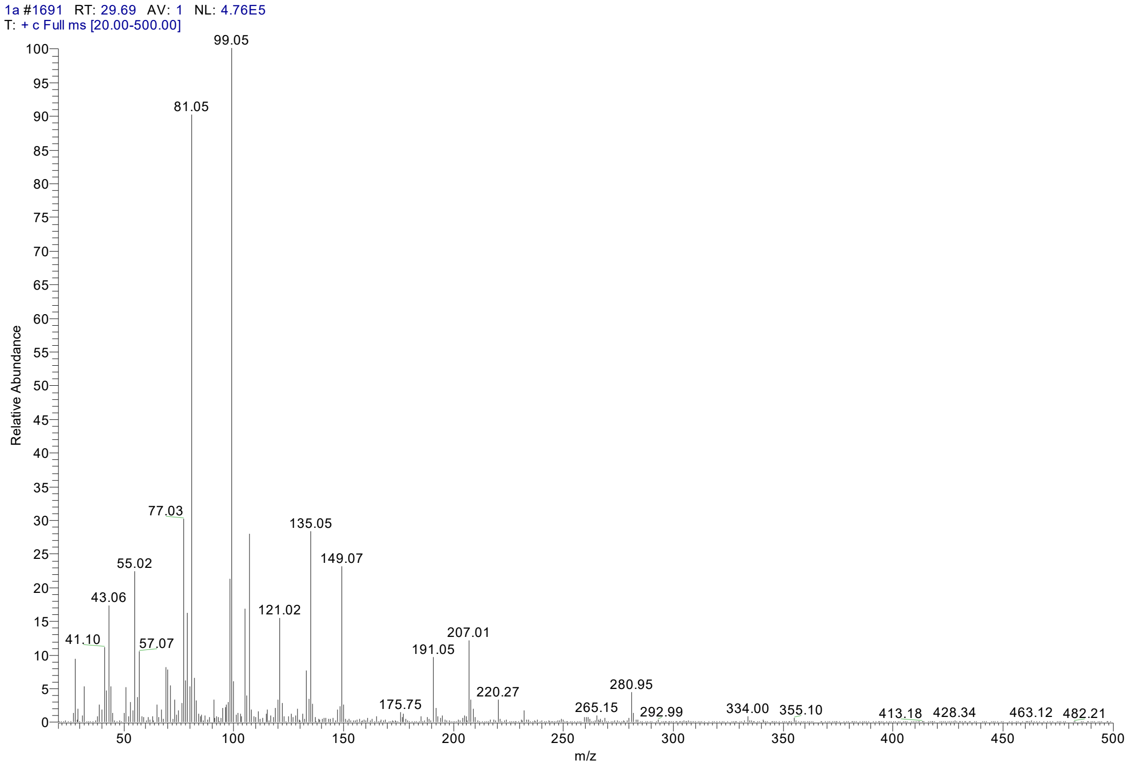

Figure S28.- SPME sampling GC-MS analysis**.** Mass spectra of peak at retention time 29.69 minutes.

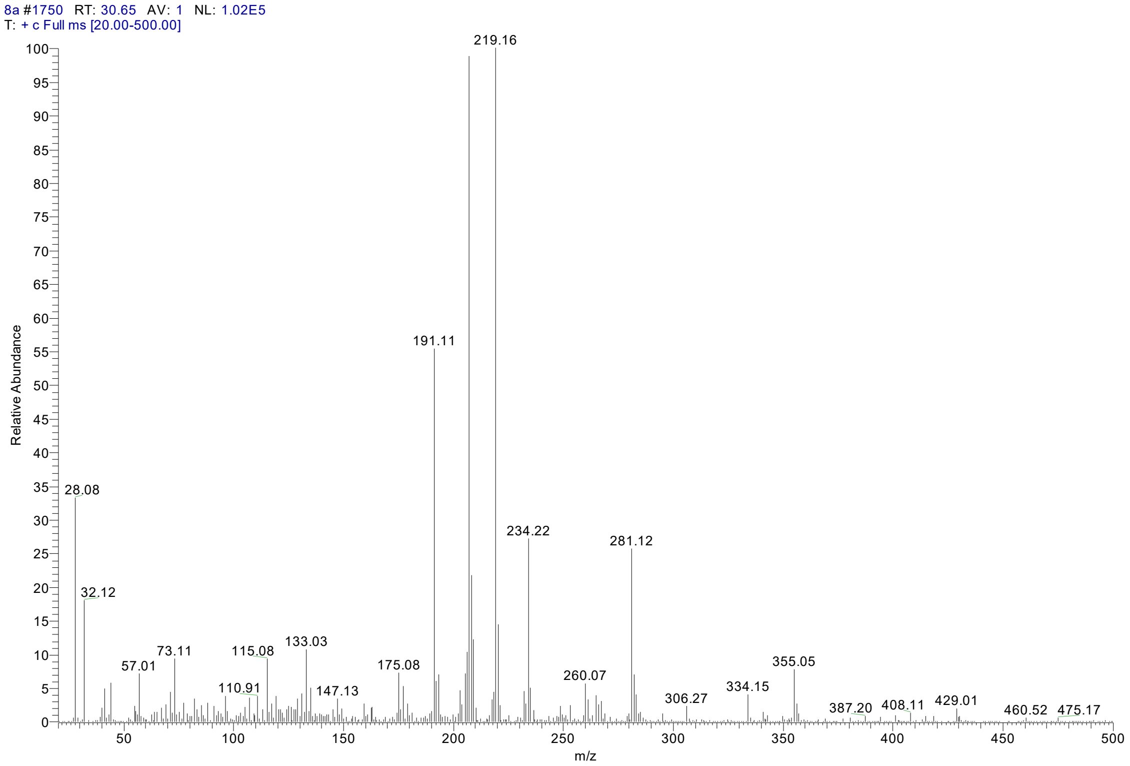

Figure S29.- SPME sampling GC-MS analysis**.** Mass spectra of peak at retention time 30.65 minutes.

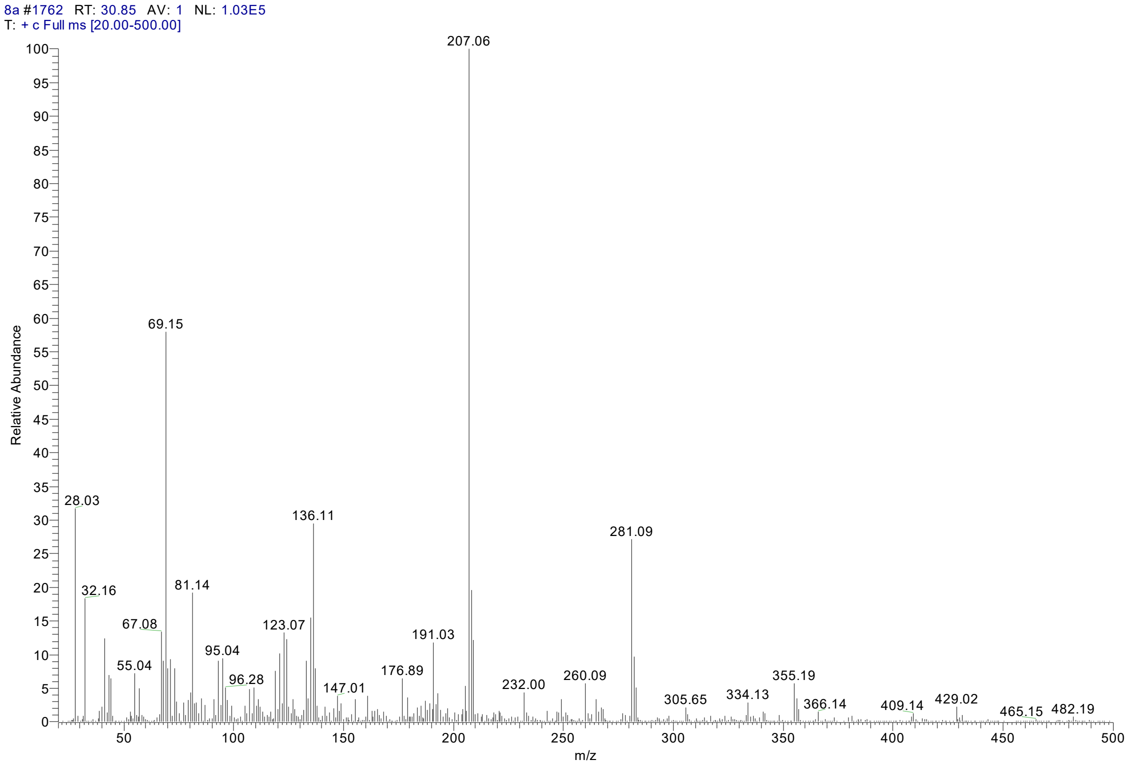

Figure S30.- SPME sampling GC-MS analysis**.** Mass spectra of peak at retention time 30.85 minutes.
